# Supplementary material for: Design, Synthesis, and Antiproliferative Activity of New 5-Chloro-indole-2-carboxylate and Pyrrolo[3,4-b]indol-3-one Derivatives as Potent Inhibitors of EGFRT790M/BRAFV600E Pathways
Source: Molecules. 2023 Jan 28;28(3):1269. doi: 10.3390/molecules28031269 (PMC9921301; doi:10.3390/molecules28031269)
Supplement: Supplementary file 1 [file molecules-28-01269-s001.zip › molecules-2180230-SI.pdf]

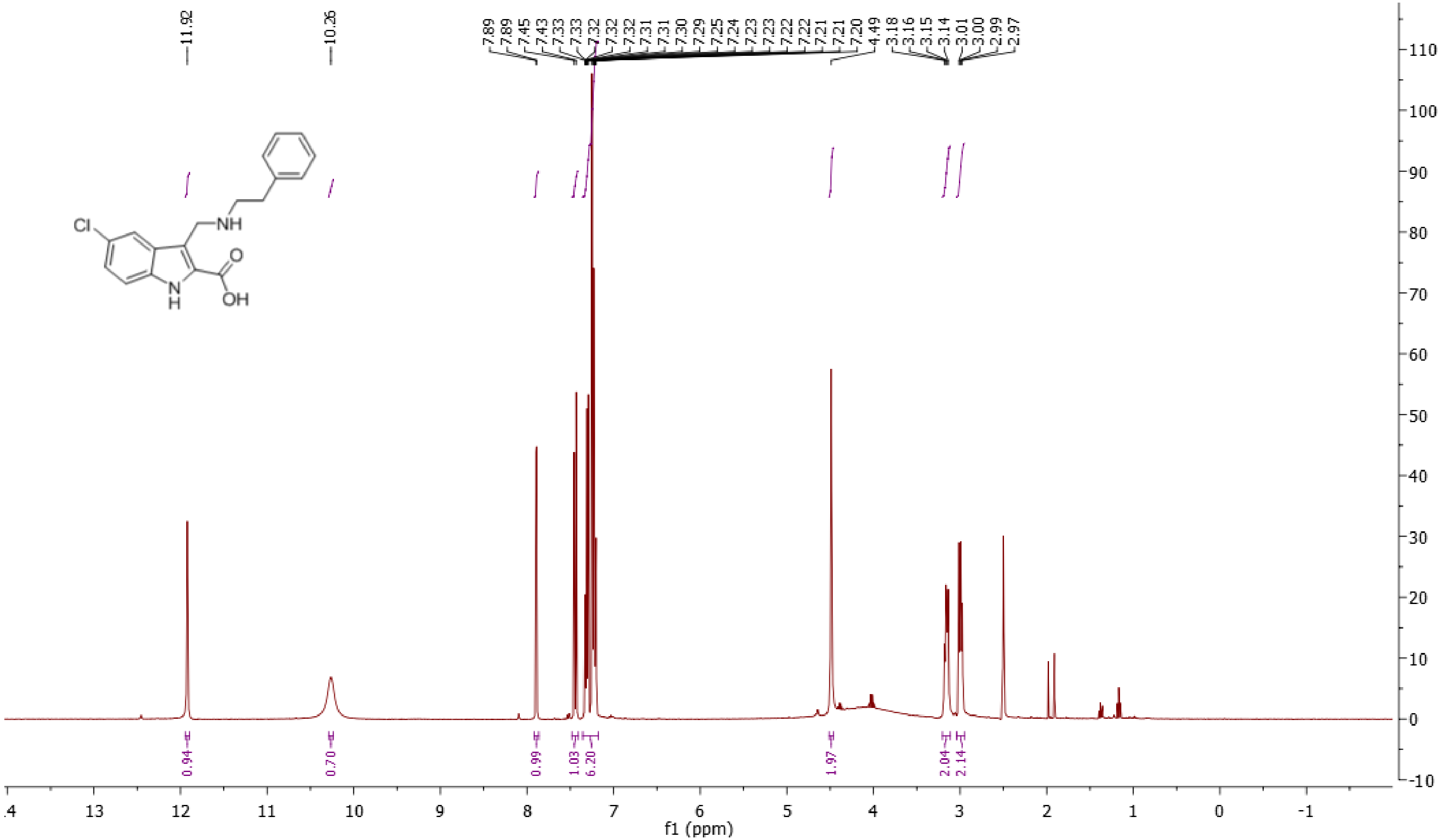

^{1}H NMR (400 MHz, DMSO-d_6) δ 11.92 (s, 1H), 10.26 (s, 1H), 7.89 (d, J = 2.0 Hz, 1H), 7.44 (d, J = 8.7 Hz, 1H), 7.36 – 7.18 (m, 6H), 4.49 (s, 2H), 3.16 (t, J = 6.2 Hz, 2H), 2.99 (t, J = 6.2 Hz, 2H).

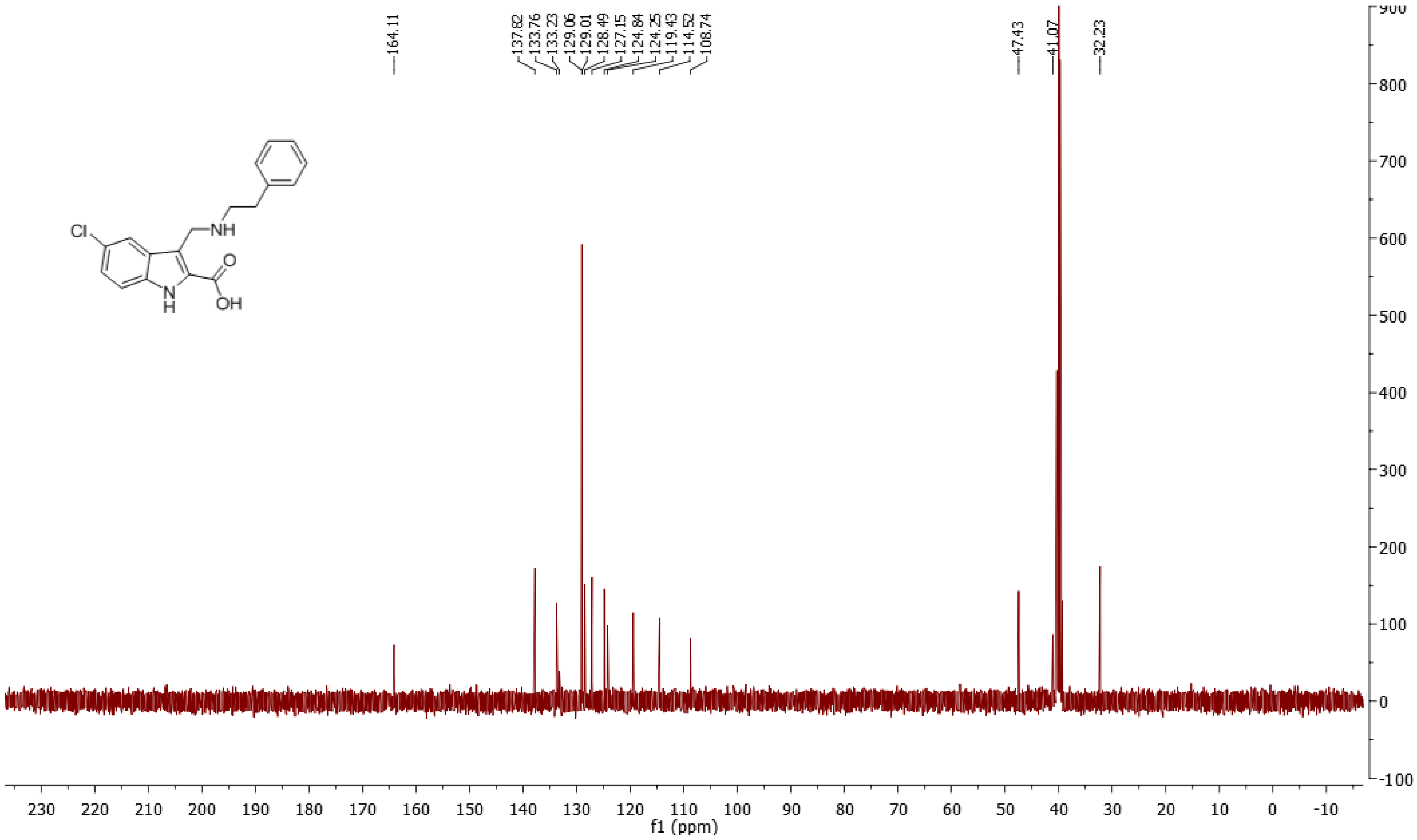

^{13}C NMR (101 MHz, dmso) δ 164.11, 137.82, 133.76, 133.23, 129.06, 129.01, 128.49, 127.15, 124.84, 124.25, 119.43, 114.52, 108.74, 47.43, 41.07, 32.23.

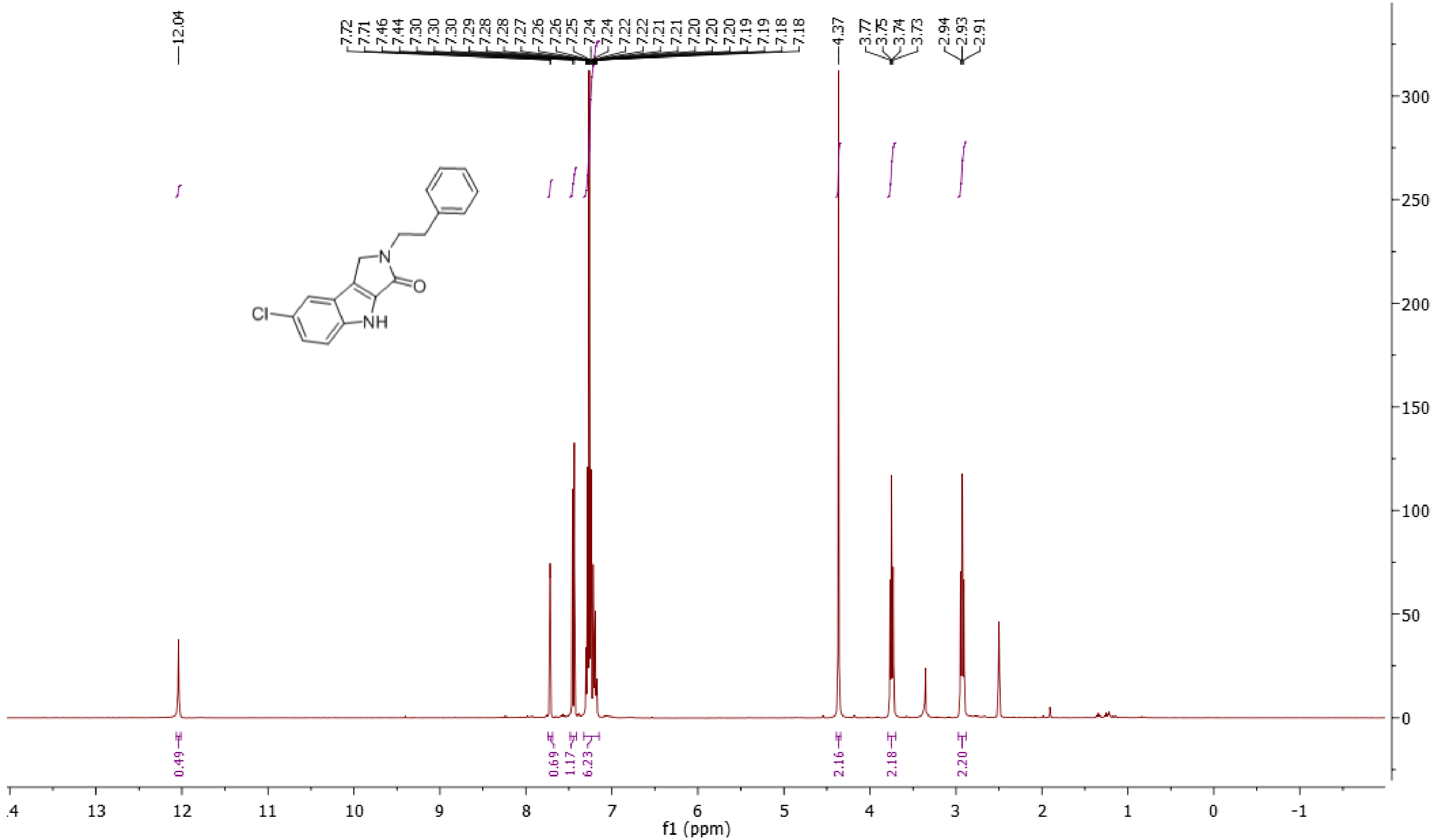

^{1}H NMR (400 MHz, DMSO-d_6) δ 12.04 (s, 1H), 7.72 (d, J = 2.1 Hz, 1H), 7.45 (d, J = 8.8 Hz, 1H), 7.33 – 7.15 (m, 6H), 4.37 (s, 2H), 3.75 (t, J = 7.3 Hz, 2H), 2.93 (t, J = 7.3 Hz, 2H).

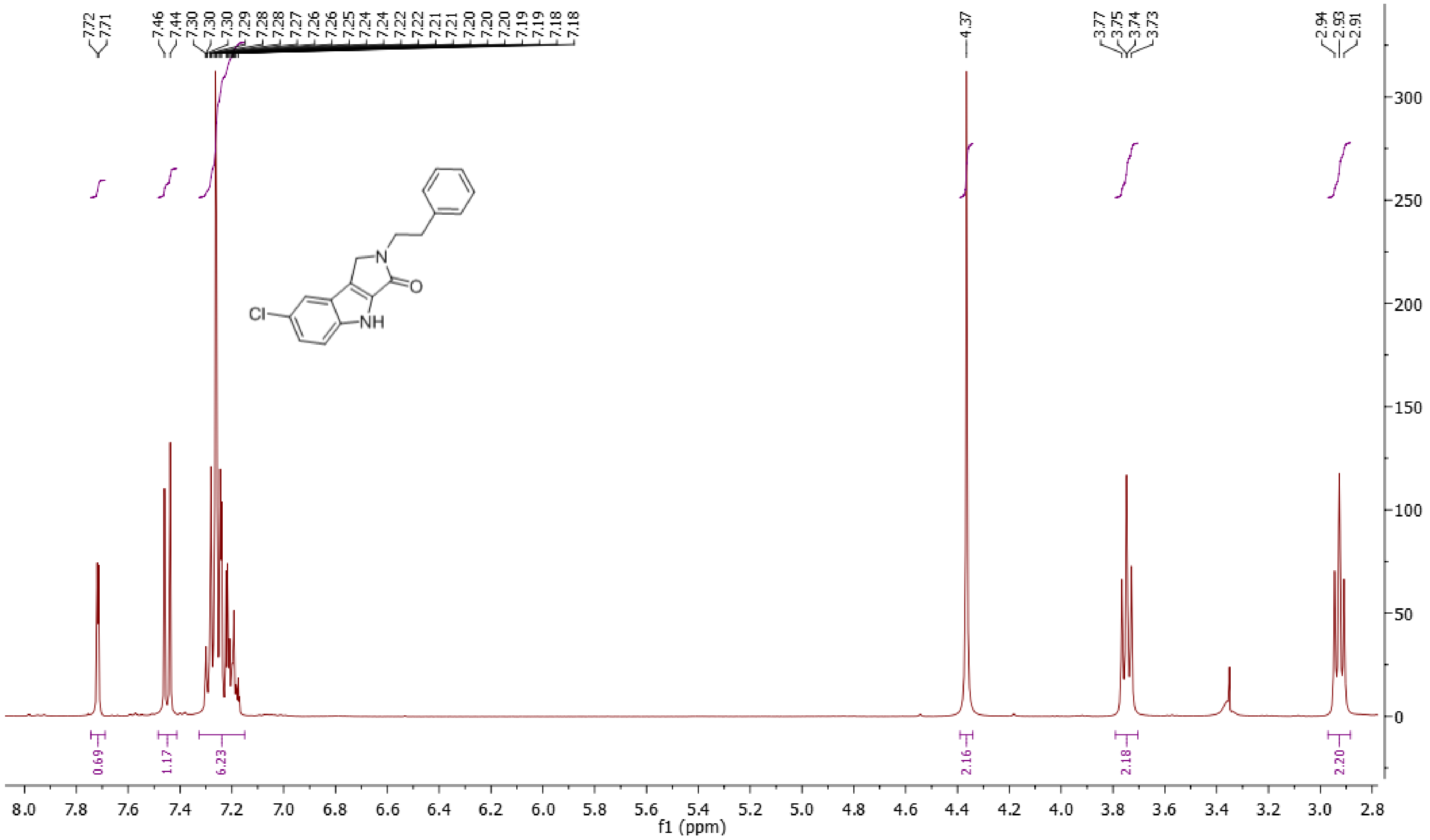

^{1}H NMR (400 MHz, DMSO-d_6) δ 12.04 (s, 1H), 7.72 (d, J = 2.1 Hz, 1H), 7.45 (d, J = 8.8 Hz, 1H), 7.33 – 7.15 (m, 6H), 4.37 (s, 2H), 3.75 (t, J = 7.3 Hz, 2H), 2.93 (t, J = 7.3 Hz, 2H).

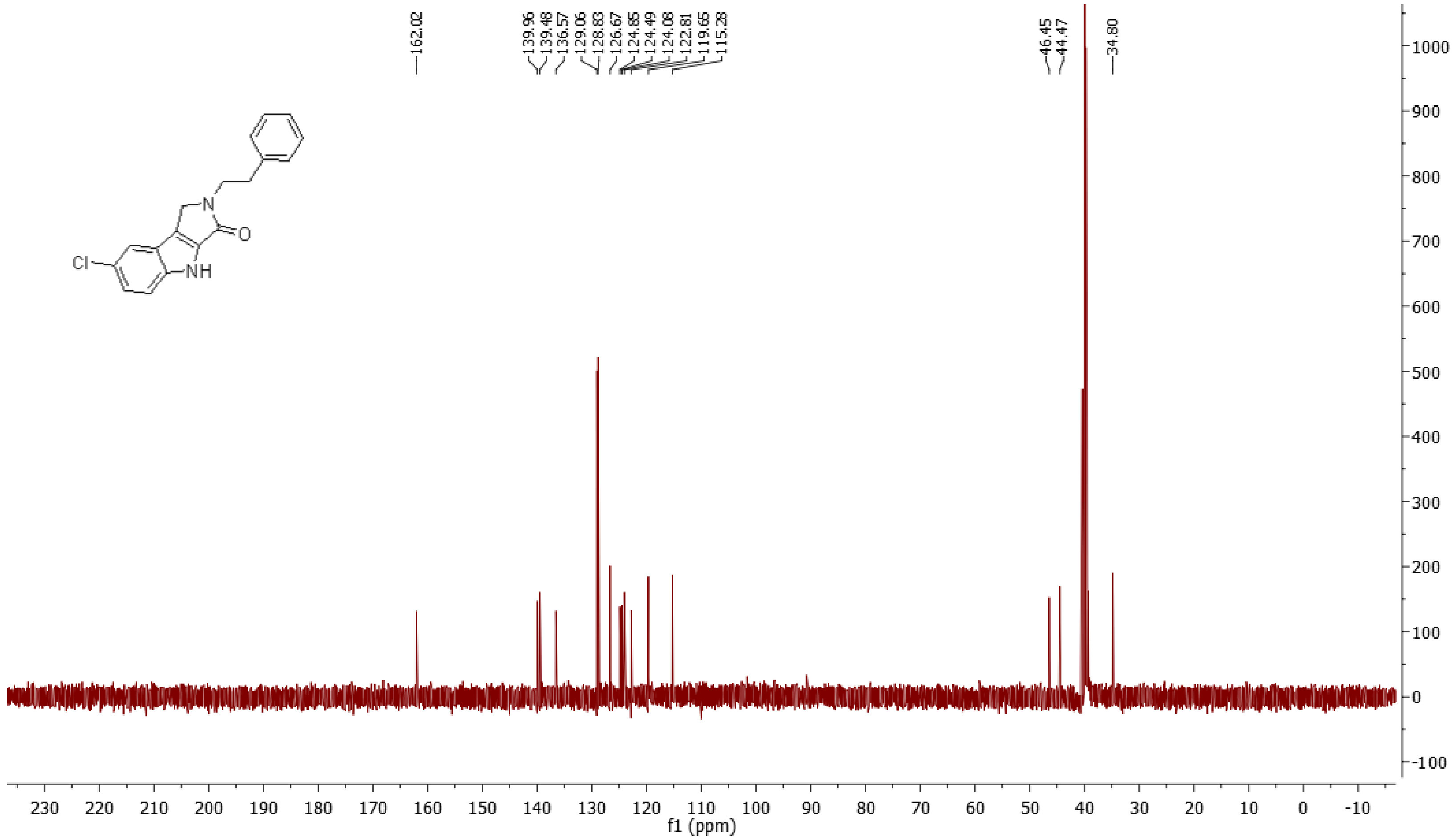

^{13}C NMR (101 MHz, dmso) δ 162.02, 139.96, 139.48, 136.57, 129.06, 128.83, 126.67, 124.85, 124.49, 124.08, 122.81, 119.65, 115.28, 46.45, 44.47, 34.80.

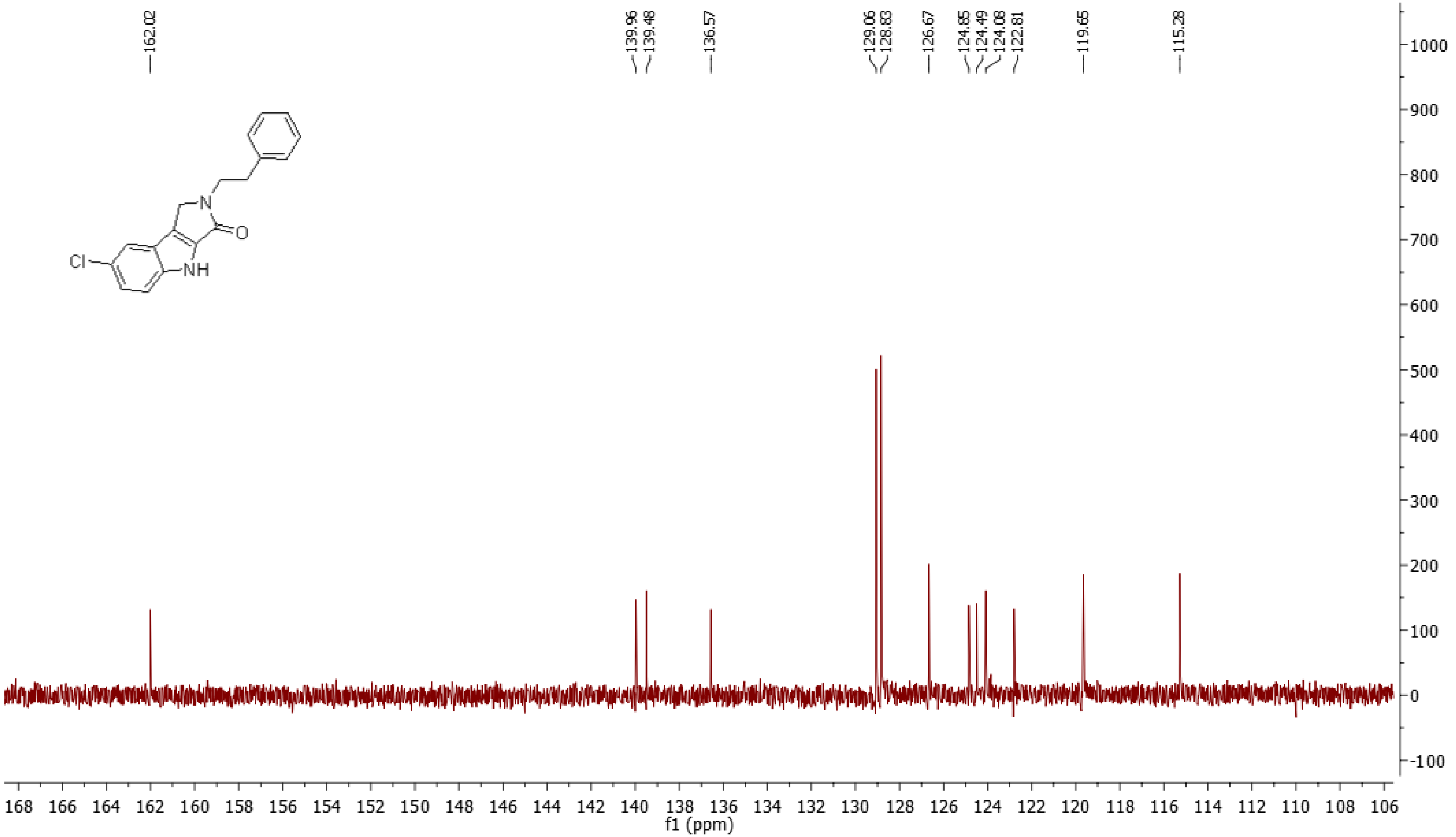

^{13}C NMR (101 MHz, dmso) δ 162.02, 139.96, 139.48, 136.57, 129.06, 128.83, 126.67, 124.85, 124.49, 124.08, 122.81, 119.65, 115.28, 46.45, 44.47, 34.80.

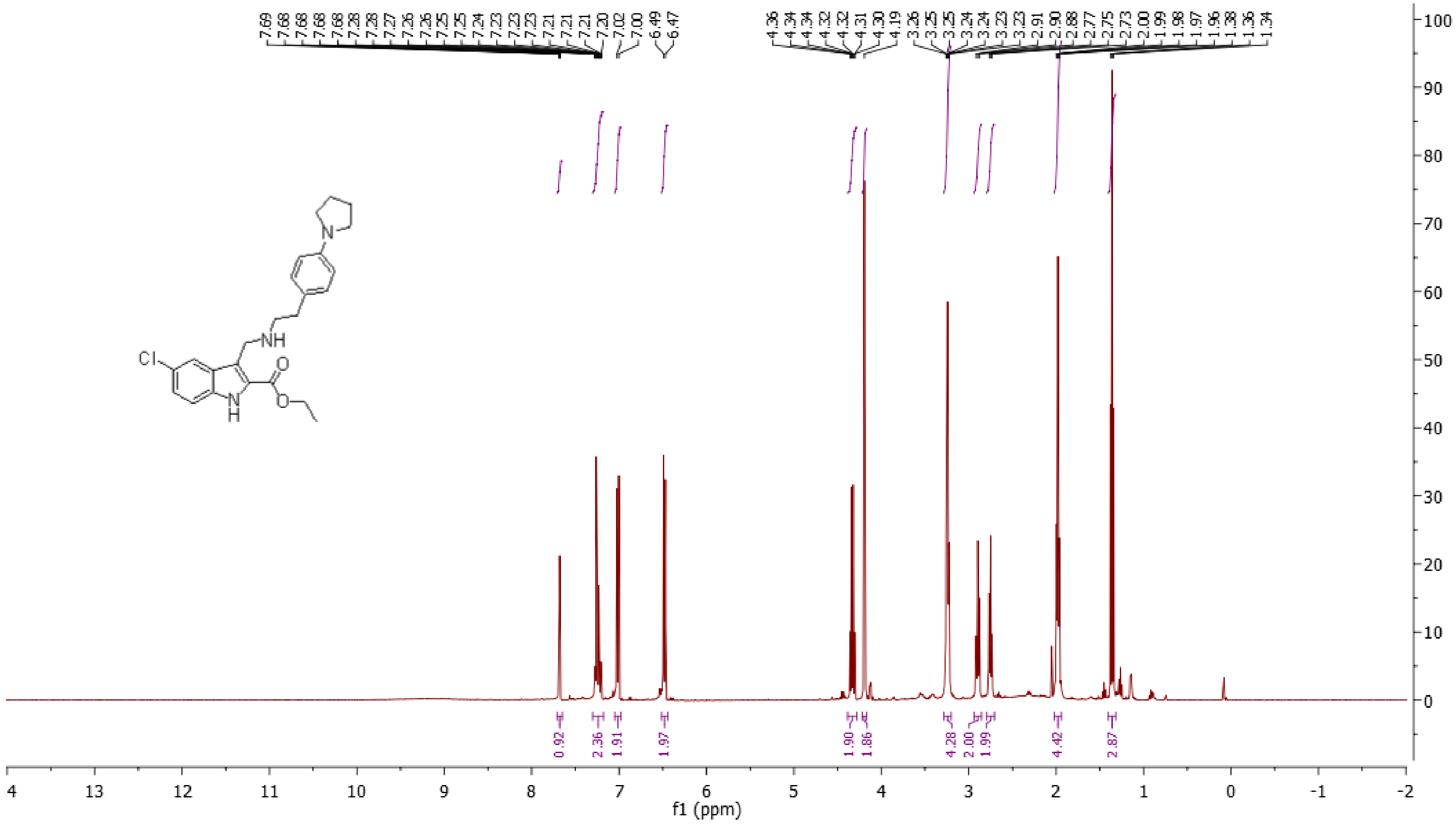

^{1}H NMR (400 MHz, Chloroform-*d*) δ 7.68 (d, *J* = 1.9 Hz, 1H), 7.30 – 7.18 (m, 2H), 7.01 (d, *J* = 8.5 Hz, 2H), 6.48 (d, *J* = 8.5 Hz, 2H), 4.33 (q, *J* = 7.2 Hz, 2H), 4.19 (s, 2H), 3.28 – 3.20 (m, 4H), 2.90 (t, *J* = 7.1 Hz, 2H), 2.75 (t, *J* = 7.1 Hz, 2H), 2.02 – 1.94 (m, 4H), 1.36 (t, *J* = 7.1 Hz, 3H).

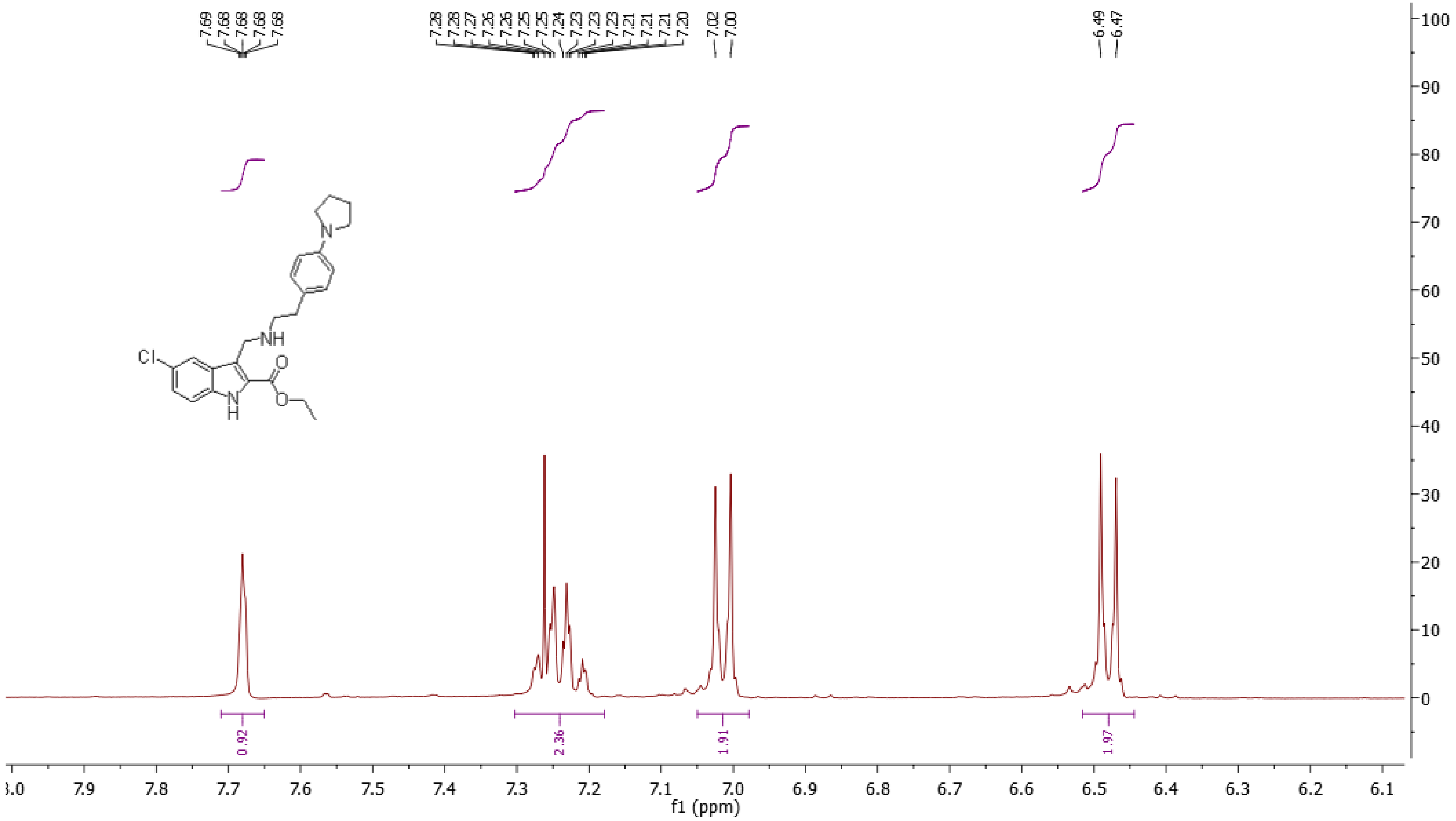

^{1}H NMR (400 MHz, Chloroform-*d*) δ 7.68 (d, *J* = 1.9 Hz, 1H), 7.30 – 7.18 (m, 2H), 7.01 (d, *J* = 8.5 Hz, 2H), 6.48 (d, *J* = 8.5 Hz, 2H), 4.33 (q, *J* = 7.2 Hz, 2H), 4.19 (s, 2H), 3.28 – 3.20 (m, 4H), 2.90 (t, *J* = 7.1 Hz, 2H), 2.75 (t, *J* = 7.1 Hz, 2H), 2.02 – 1.94 (m, 4H), 1.36 (t, *J* = 7.1 Hz, 3H).

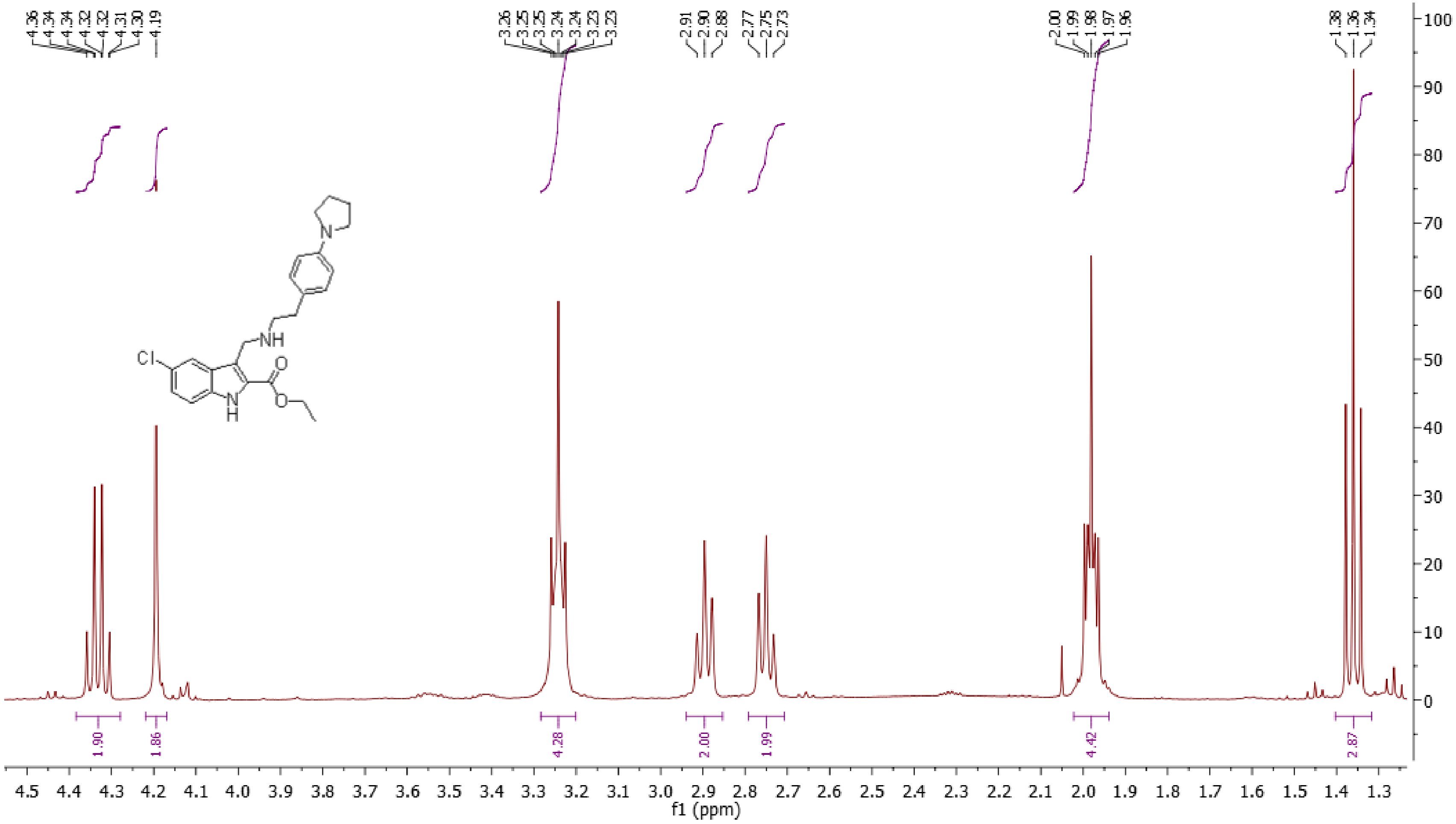

^{1}H NMR (400 MHz, Chloroform-*d*) δ 7.68 (d, *J* = 1.9 Hz, 1H), 7.30 – 7.18 (m, 2H), 7.01 (d, *J* = 8.5 Hz, 2H), 6.48 (d, *J* = 8.5 Hz, 2H), 4.33 (q, *J* = 7.2 Hz, 2H), 4.19 (s, 2H), 3.28 – 3.20 (m, 4H), 2.90 (t, *J* = 7.1 Hz, 2H), 2.75 (t, *J* = 7.1 Hz, 2H), 2.02 – 1.94 (m, 4H), 1.36 (t, *J* = 7.1 Hz, 3H).

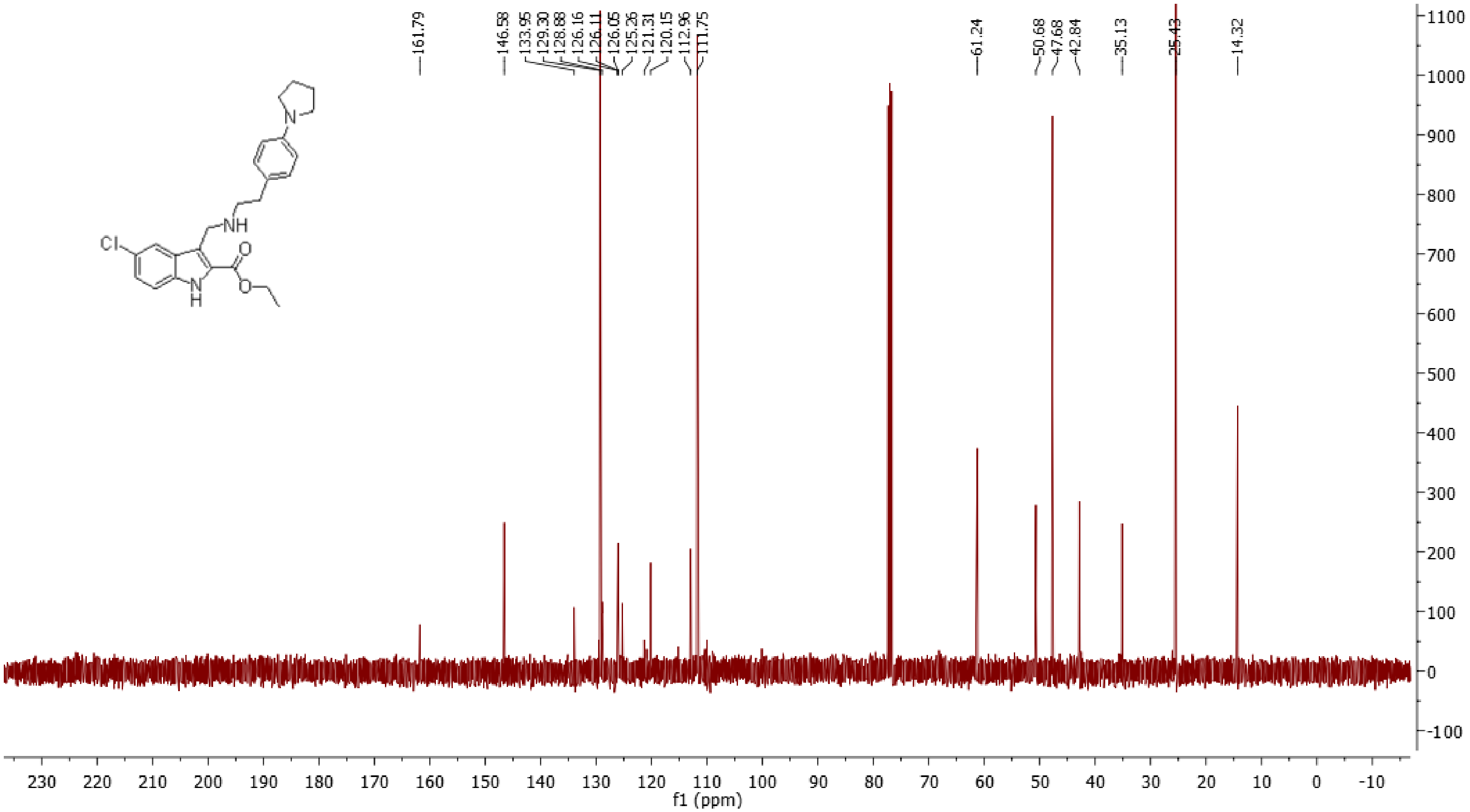

^{13}C NMR (101 MHz, cdcl$_3$) δ 161.79, 146.58, 133.95, 129.30, 128.88, 126.16, 126.11, 126.05, 125.26, 121.31, 120.15, 112.96, 111.75, 61.24, 50.68, 47.68, 42.84, 35.13, 25.43, 14.32.

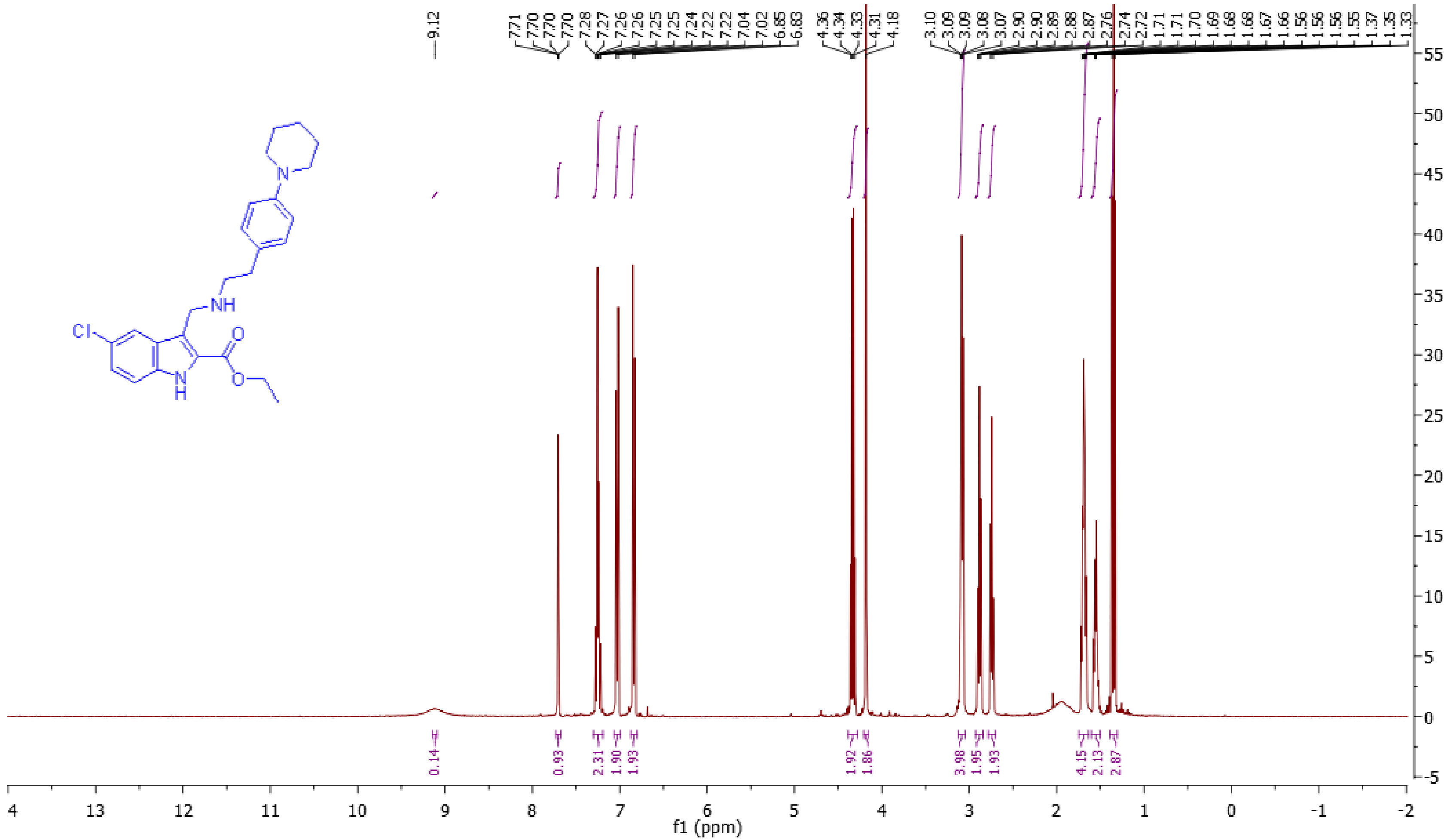

^{1}H NMR (400 MHz, Chloroform-*d*) δ 9.12 (s, 1H), 7.70 (d, *J* = 1.8 Hz, 1H), 7.30 – 7.19 (m, 2H), 7.03 (d, *J* = 8.5 Hz, 2H), 6.84 (d, *J* = 8.6 Hz, 2H), 4.33 (q, *J* = 7.1 Hz, 2H), 4.18 (s, 2H), 3.13 – 3.05 (m, 4H), 2.88 (t, *J* = 7.1 Hz, 2H), 2.74 (t, *J* = 7.1 Hz, 2H), 1.72 - 1.65 (m, 4H), 1.58 - 1.52 (m, 2H), 1.35 (t, *J* = 7.1 Hz, 3H).

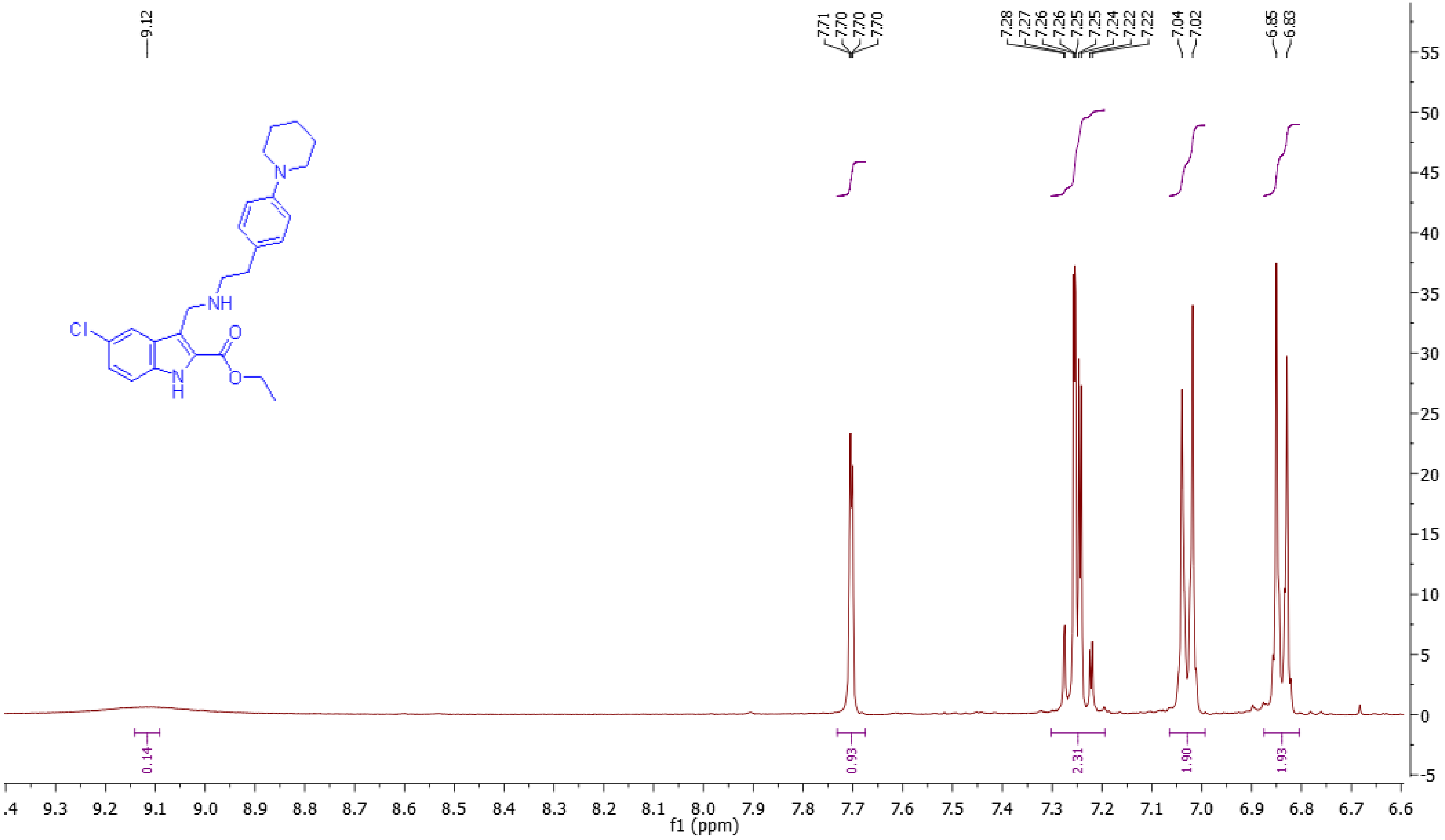

^{1}H NMR (400 MHz, Chloroform-*d*) δ 9.12 (s, 1H), 7.70 (d, *J* = 1.8 Hz, 1H), 7.30 – 7.19 (m, 2H), 7.03 (d, *J* = 8.5 Hz, 2H), 6.84 (d, *J* = 8.6 Hz, 2H), 4.33 (q, *J* = 7.1 Hz, 2H), 4.18 (s, 2H), 3.13 – 3.05 (m, 4H), 2.88 (t, *J* = 7.1 Hz, 2H), 2.74 (t, *J* = 7.1 Hz, 2H), 1.72 - 1.65 (m, 4H), 1.58 - 1.52 (m, 2H), 1.35 (t, *J* = 7.1 Hz, 3H).

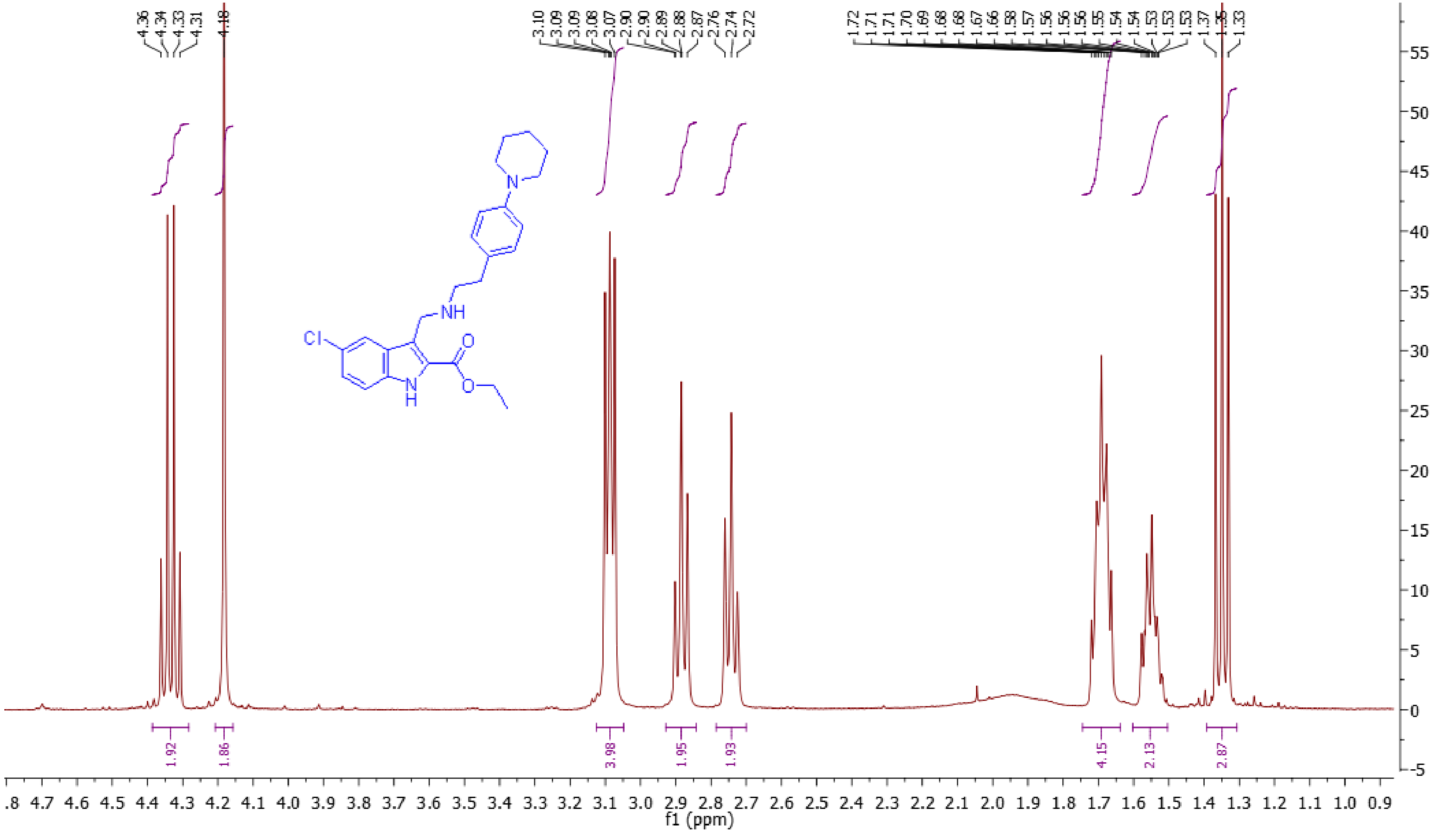

^{1}H NMR (400 MHz, Chloroform-*d*) δ 9.12 (s, 1H), 7.70 (d, *J* = 1.8 Hz, 1H), 7.30 – 7.19 (m, 2H), 7.03 (d, *J* = 8.5 Hz, 2H), 6.84 (d, *J* = 8.6 Hz, 2H), 4.33 (q, *J* = 7.1 Hz, 2H), 4.18 (s, 2H), 3.13 – 3.05 (m, 4H), 2.88 (t, *J* = 7.1 Hz, 2H), 2.74 (t, *J* = 7.1 Hz, 2H), 1.72 - 1.65 (m, 4H), 1.58 - 1.52 (m, 2H), 1.35 (t, *J* = 7.1 Hz, 3H).

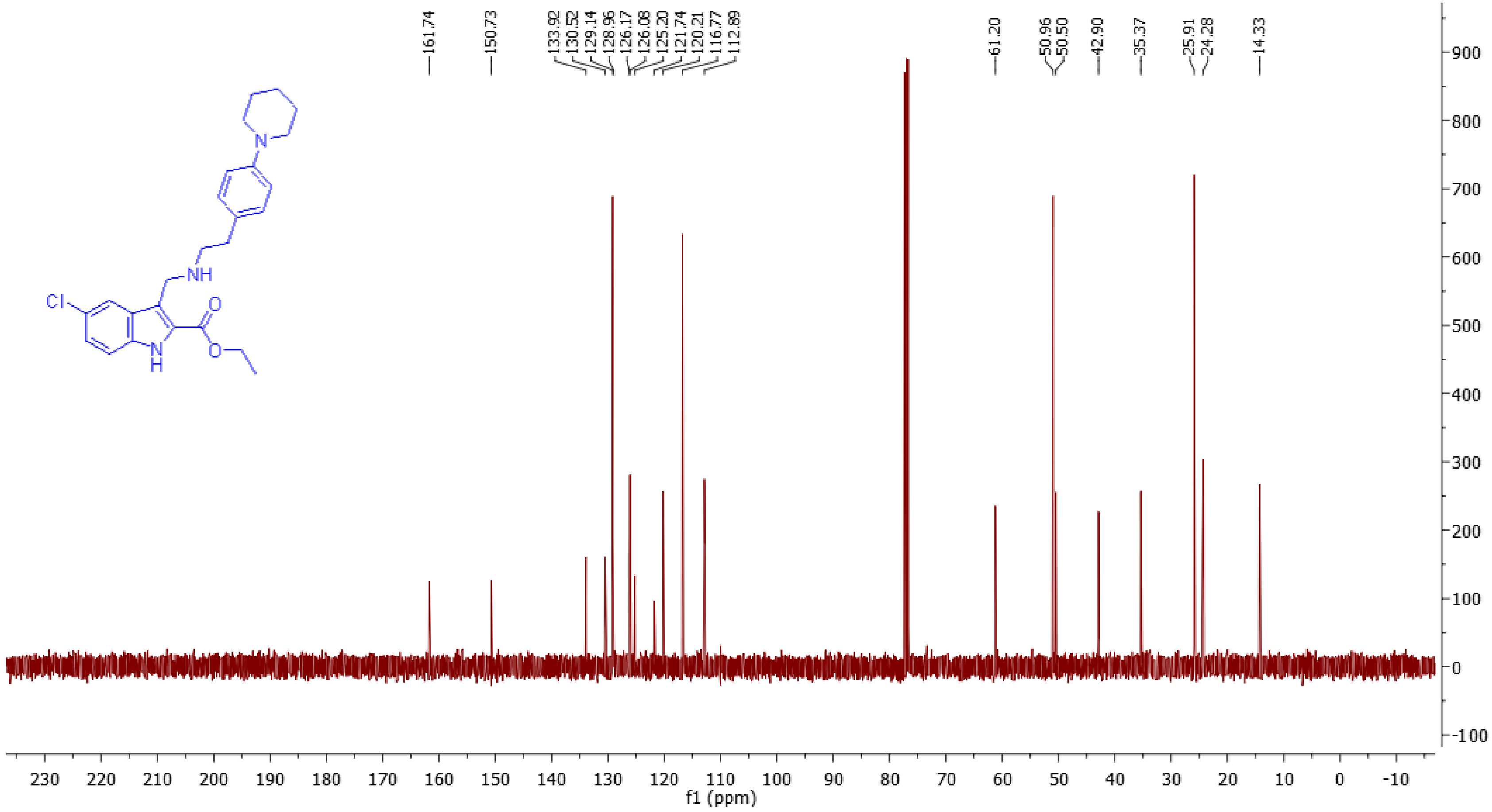

^{13}C NMR (101 MHz, cdcl$_3$) δ 161.74, 150.73, 133.92, 130.52, 129.14, 128.96, 126.17, 126.08, 125.20, 121.74, 120.21, 116.77, 112.89, 61.20, 50.96, 50.50, 42.90, 35.37, 25.91, 24.28, 14.33.

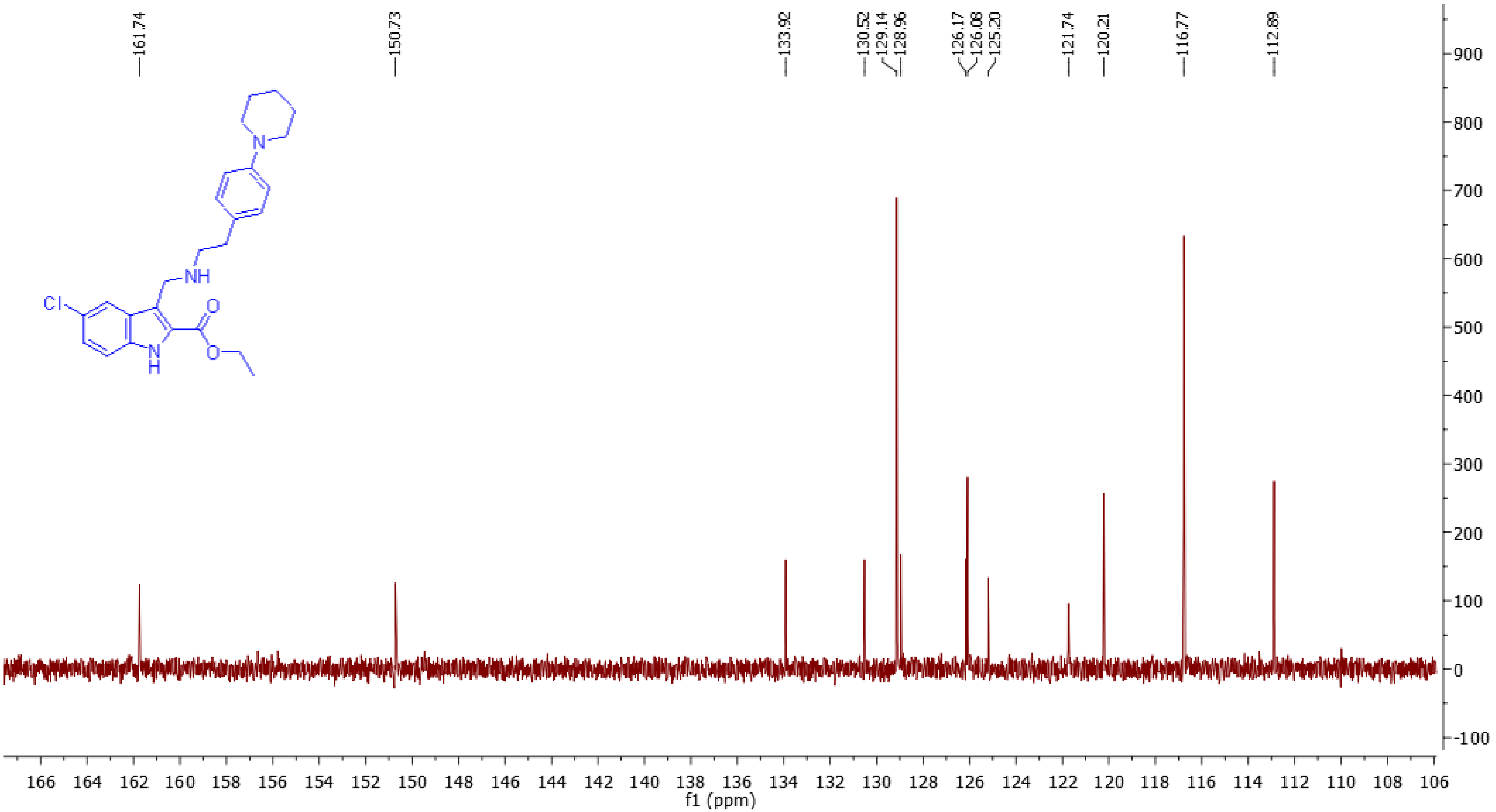

^{13}C NMR (101 MHz, cdcl$_3$) δ 161.74, 150.73, 133.92, 130.52, 129.14, 128.96, 126.17, 126.08, 125.20, 121.74, 120.21, 116.77, 112.89, 61.20, 50.96, 50.50, 42.90, 35.37, 25.91, 24.28, 14.33.

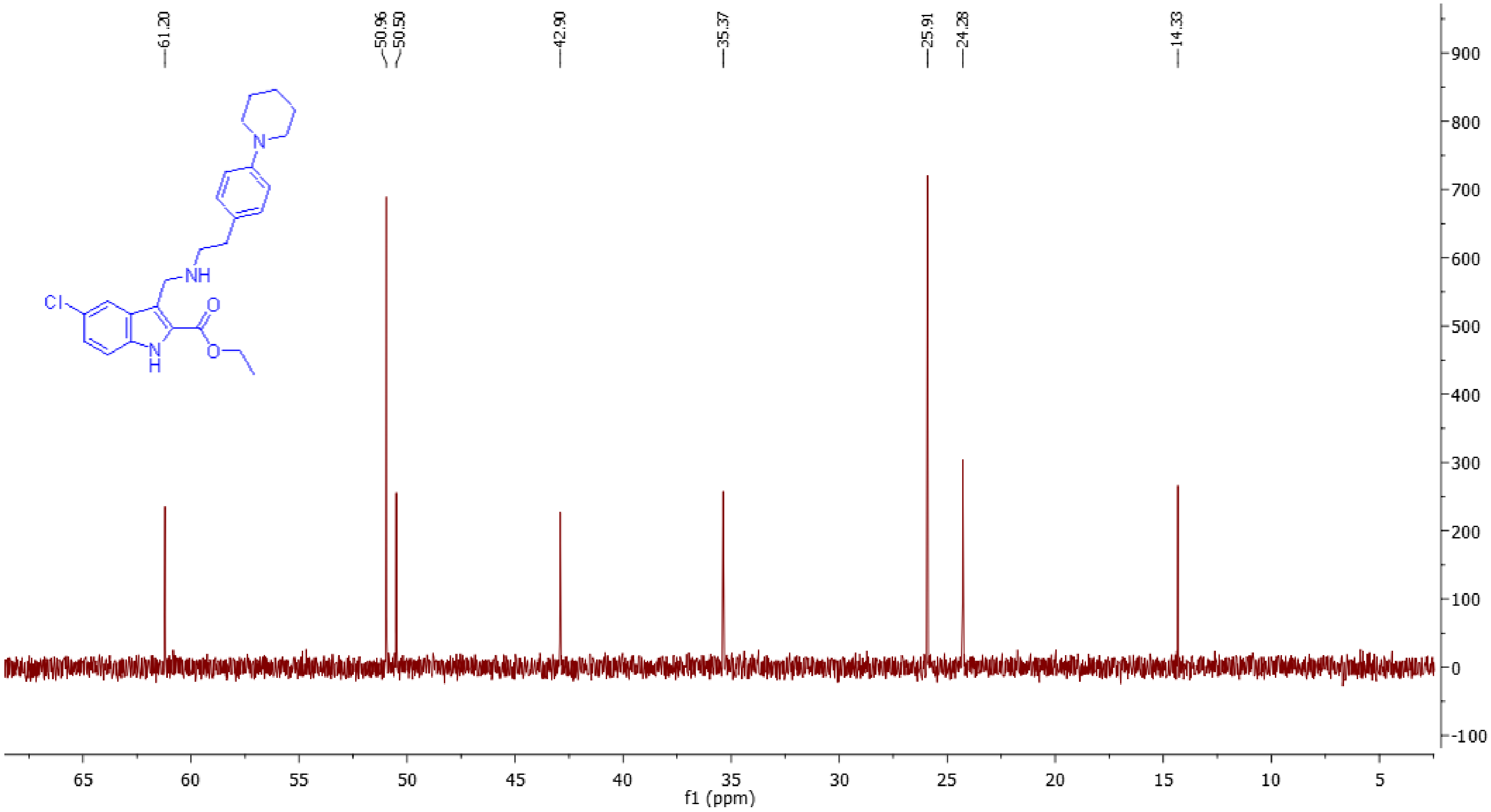

^{13}C NMR (101 MHz, cdcl$_3$) δ 161.74, 150.73, 133.92, 130.52, 129.14, 128.96, 126.17, 126.08, 125.20, 121.74, 120.21, 116.77, 112.89, 61.20, 50.96, 50.50, 42.90, 35.37, 25.91, 24.28, 14.33.

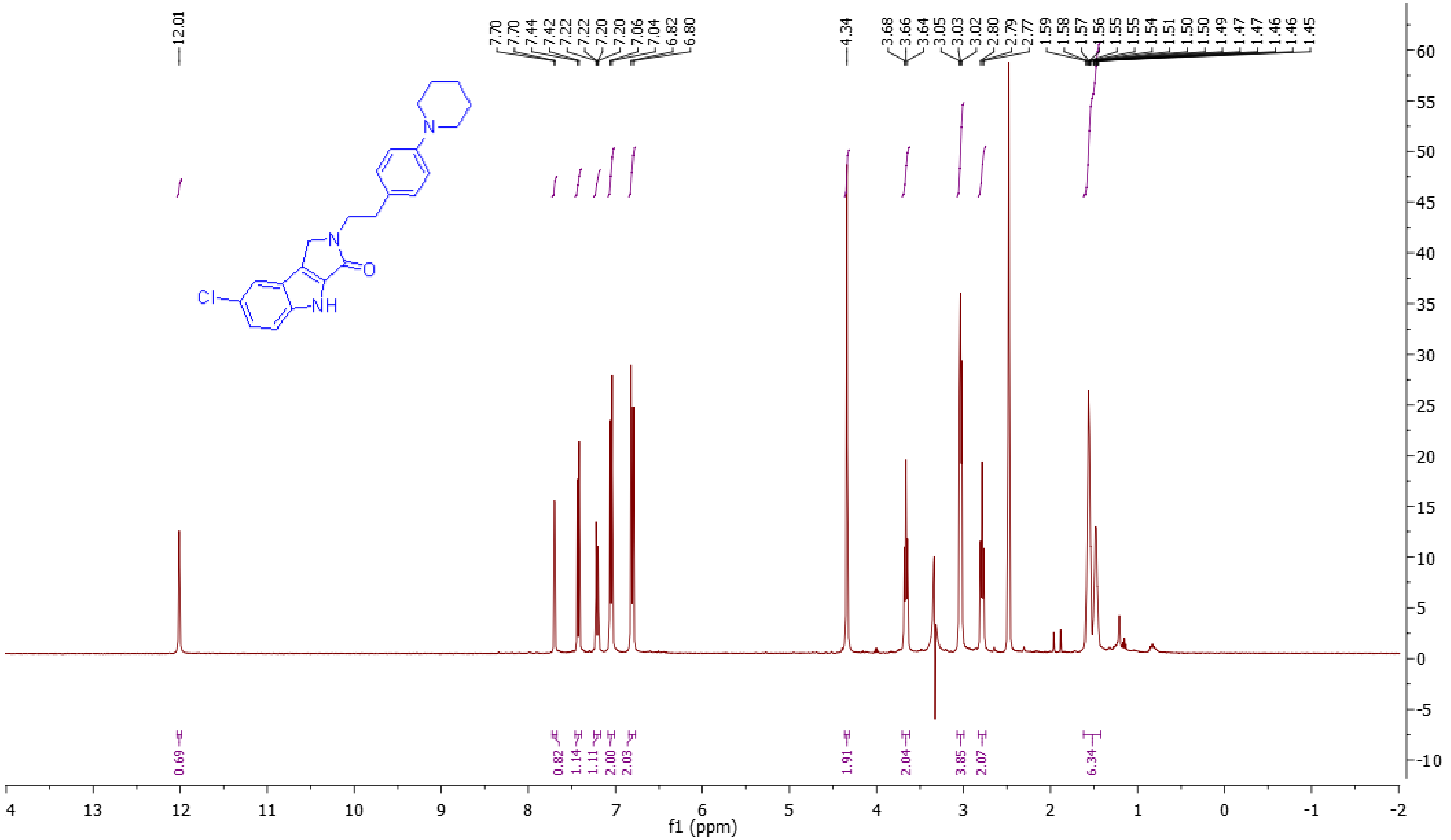

^{1}H NMR (400 MHz, DMSO-d_6) δ 12.01 (s, 1H), 7.70 (d, J = 2.1 Hz, 1H), 7.43 (d, J = 8.8 Hz, 1H), 7.21 (dd, J = 8.8, 2.2 Hz, 1H), 7.05 (d, J = 8.5 Hz, 2H), 6.81 (d, J = 8.5 Hz, 2H), 4.34 (s, 2H), 3.66 (t, J = 7.3 Hz, 2H), 3.07 – 2.99 (m, 4H), 2.79 (t, J = 7.3 Hz, 2H), 1.62 – 1.42 (m, 6H).

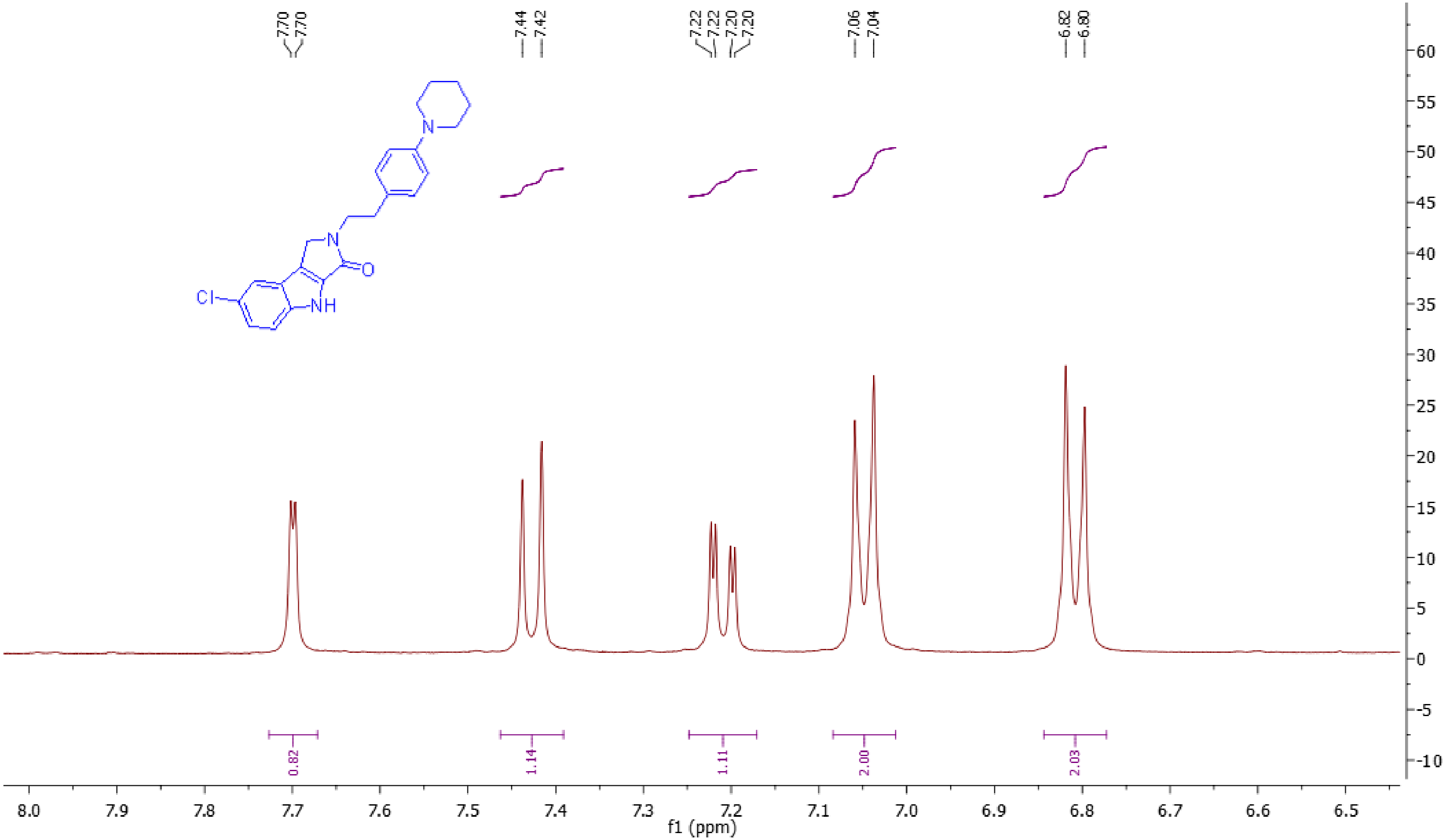

^{1}H NMR (400 MHz, DMSO-d_6) δ 12.01 (s, 1H), 7.70 (d, J = 2.1 Hz, 1H), 7.43 (d, J = 8.8 Hz, 1H), 7.21 (dd, J = 8.8, 2.2 Hz, 1H), 7.05 (d, J = 8.5 Hz, 2H), 6.81 (d, J = 8.5 Hz, 2H), 4.34 (s, 2H), 3.66 (t, J = 7.3 Hz, 2H), 3.07 – 2.99 (m, 4H), 2.79 (t, J = 7.3 Hz, 2H), 1.62 – 1.42 (m, 6H).

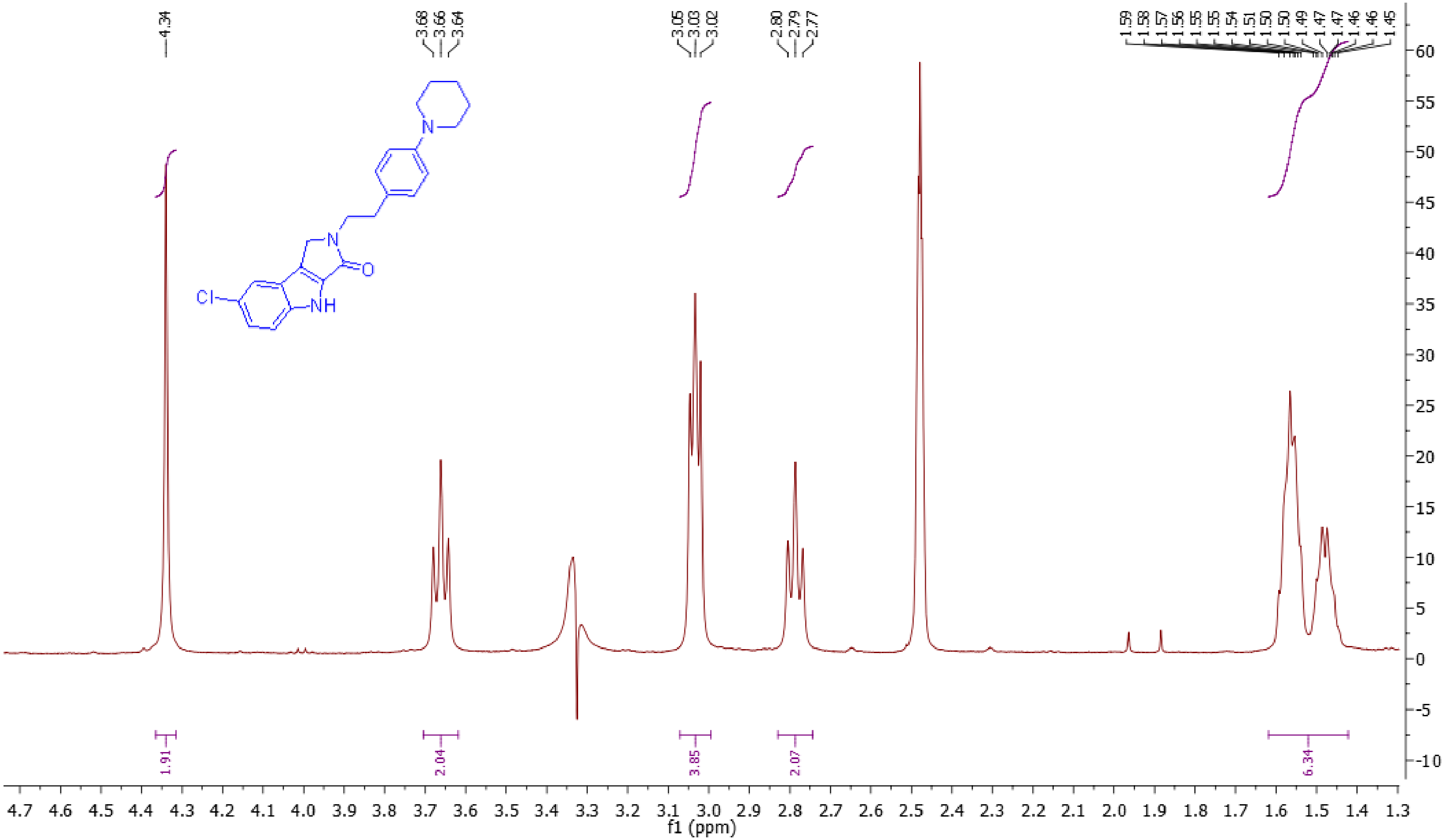

^{1}H NMR (400 MHz, DMSO-d_6) δ 12.01 (s, 1H), 7.70 (d, J = 2.1 Hz, 1H), 7.43 (d, J = 8.8 Hz, 1H), 7.21 (dd, J = 8.8, 2.2 Hz, 1H), 7.05 (d, J = 8.5 Hz, 2H), 6.81 (d, J = 8.5 Hz, 2H), 4.34 (s, 2H), 3.66 (t, J = 7.3 Hz, 2H), 3.07 – 2.99 (m, 4H), 2.79 (t, J = 7.3 Hz, 2H), 1.62 – 1.42 (m, 6H).

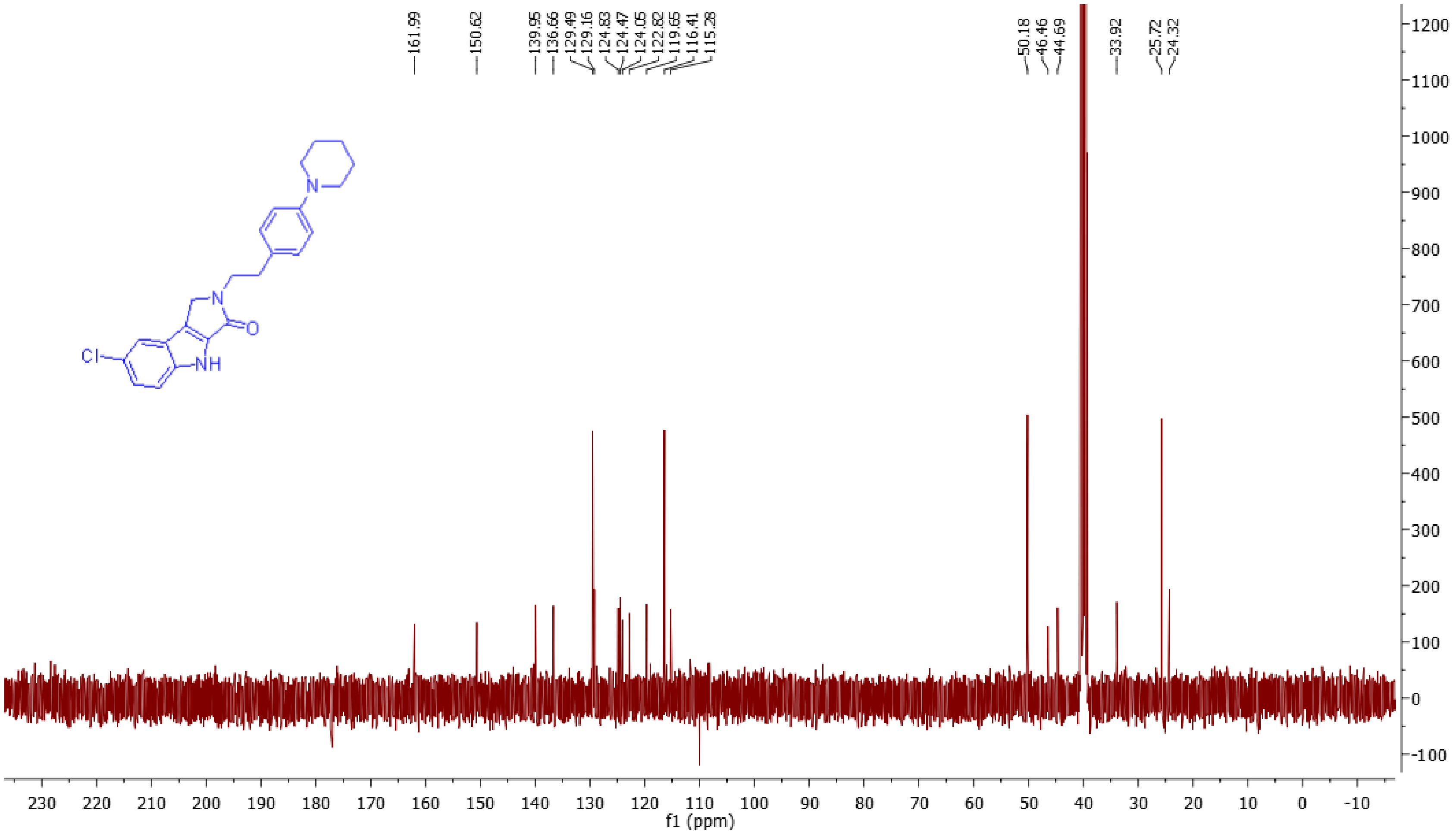

^{13}C NMR (101 MHz, dmso) δ 161.99, 150.62, 139.95, 136.66, 129.49, 129.16, 124.83, 124.47, 124.05, 122.82, 119.65, 116.41, 115.28, 50.18, 46.46, 44.69, 33.92, 25.72, 24.32.

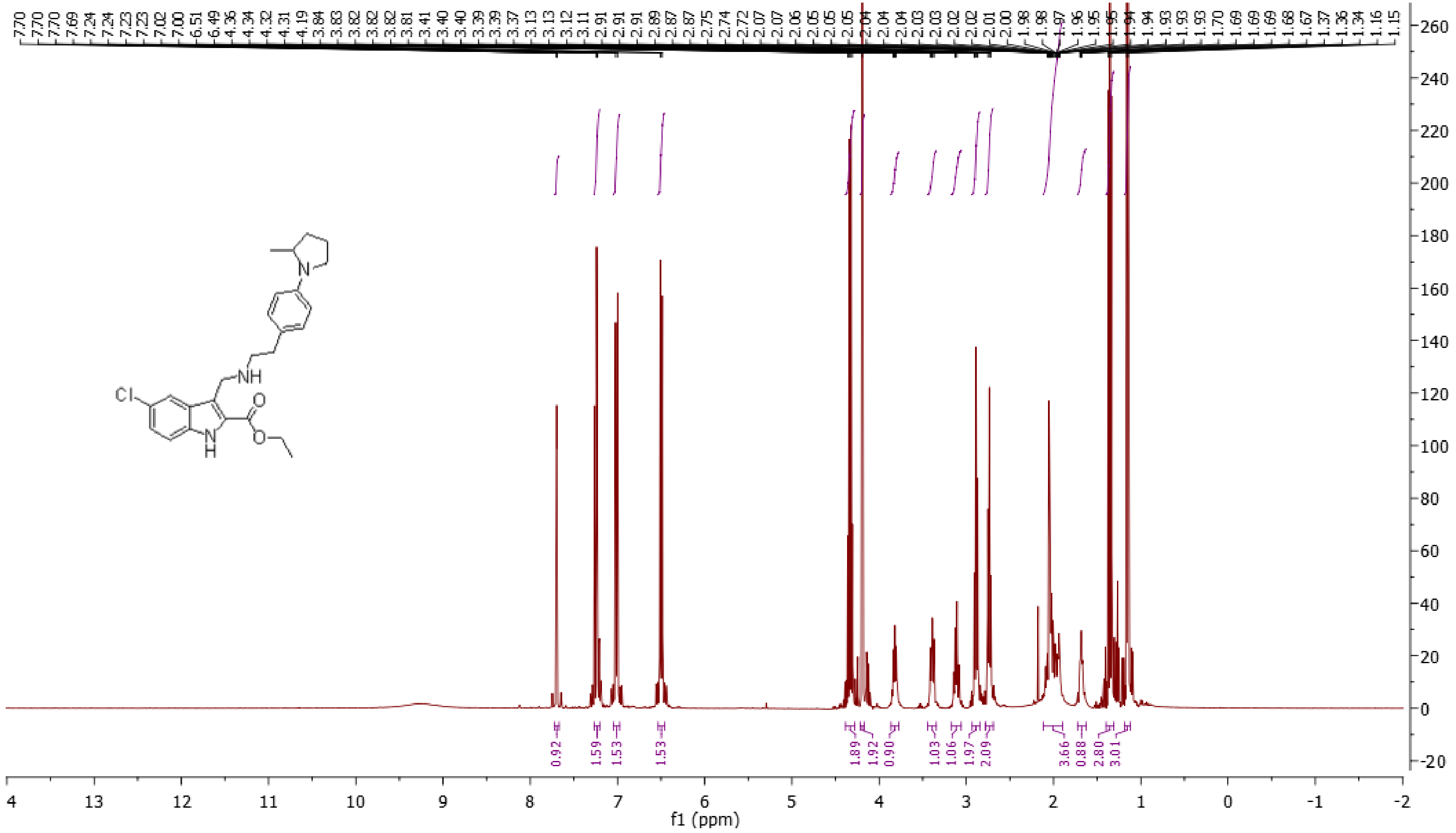

^{1}H NMR (400 MHz, Chloroform-*d*) δ 9.25 (s, 1H), 7.70 (d, *J* = 1.7 Hz, 1H), 7.24 - 2.23 (m, 2H), 7.01 (d, *J* = 8.5 Hz, 2H), 6.50 (d, *J* = 8.6 Hz, 2H), 4.33 (q, *J* = 7.1 Hz, 2H), 4.19 (s, 2H), 3.84 - 3.80 (m, 1H), 3.44 – 3.34 (m, 1H), 3.17 – 3.06 (m, 1H), 2.89 (t, *J* = 7.1 Hz, 2H), 2.74 (t, *J* = 7.1 Hz, 2H), 2.10 – 1.90 (m, 3H), 1.73 – 1.63 (m, 1H), 1.36 (t, *J* = 7.1 Hz, 3H), 1.15 (d, *J* = 6.2 Hz, 3H).

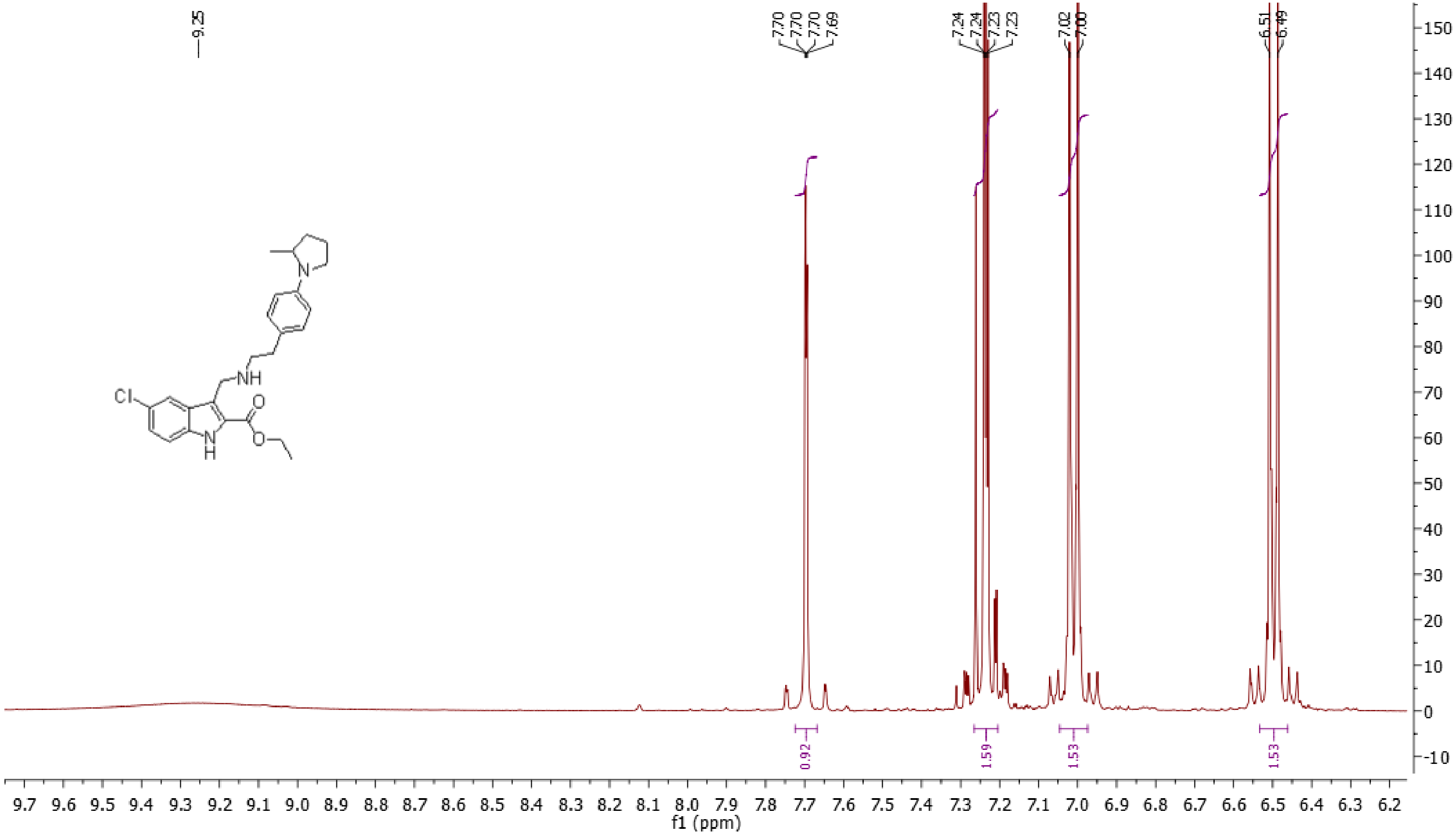

^{1}H NMR (400 MHz, Chloroform-*d*) δ 9.25 (s, 1H), 7.70 (d, *J* = 1.7 Hz, 1H), 7.24 - 2.23 (m, 2H), 7.01 (d, *J* = 8.5 Hz, 2H), 6.50 (d, *J* = 8.6 Hz, 2H), 4.33 (q, *J* = 7.1 Hz, 2H), 4.19 (s, 2H), 3.84 - 3.80 (m, 1H), 3.44 – 3.34 (m, 1H), 3.17 – 3.06 (m, 1H), 2.89 (t, *J* = 7.1 Hz, 2H), 2.74 (t, *J* = 7.1 Hz, 2H), 2.10 – 1.90 (m, 3H), 1.73 – 1.63 (m, 1H), 1.36 (t, *J* = 7.1 Hz, 3H), 1.15 (d, *J* = 6.2 Hz, 3H).

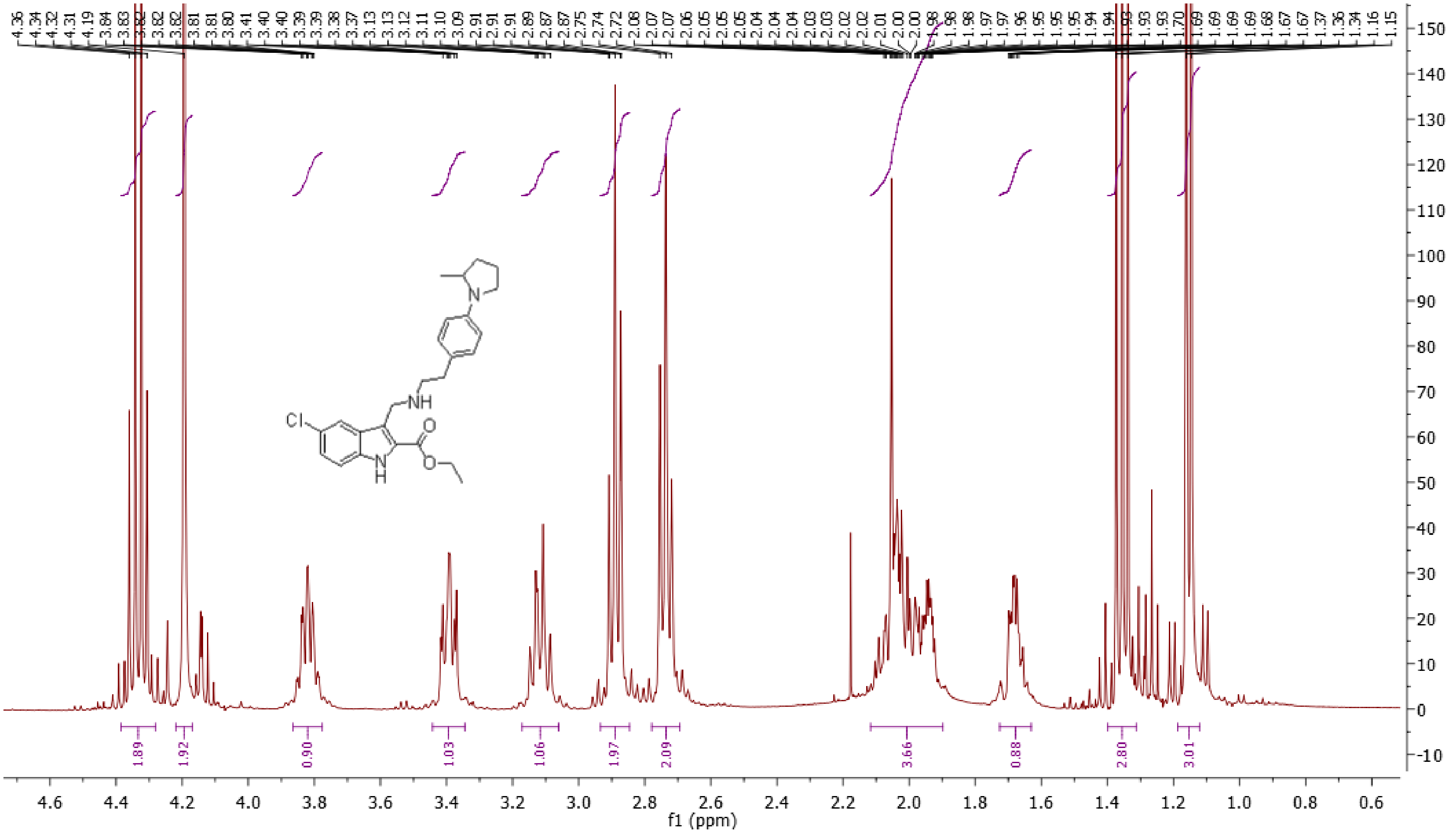

^{1}H NMR (400 MHz, Chloroform-*d*) δ 9.25 (s, 1H), 7.70 (d, *J* = 1.7 Hz, 1H), 7.24 - 2.23 (m, 2H), 7.01 (d, *J* = 8.5 Hz, 2H), 6.50 (d, *J* = 8.6 Hz, 2H), 4.33 (q, *J* = 7.1 Hz, 2H), 4.19 (s, 2H), 3.84 - 3.80 (m, 1H), 3.44 – 3.34 (m, 1H), 3.17 – 3.06 (m, 1H), 2.89 (t, *J* = 7.1 Hz, 2H), 2.74 (t, *J* = 7.1 Hz, 2H), 2.10 – 1.90 (m, 3H), 1.73 – 1.63 (m, 1H), 1.36 (t, *J* = 7.1 Hz, 3H), 1.15 (d, *J* = 6.2 Hz, 3H).

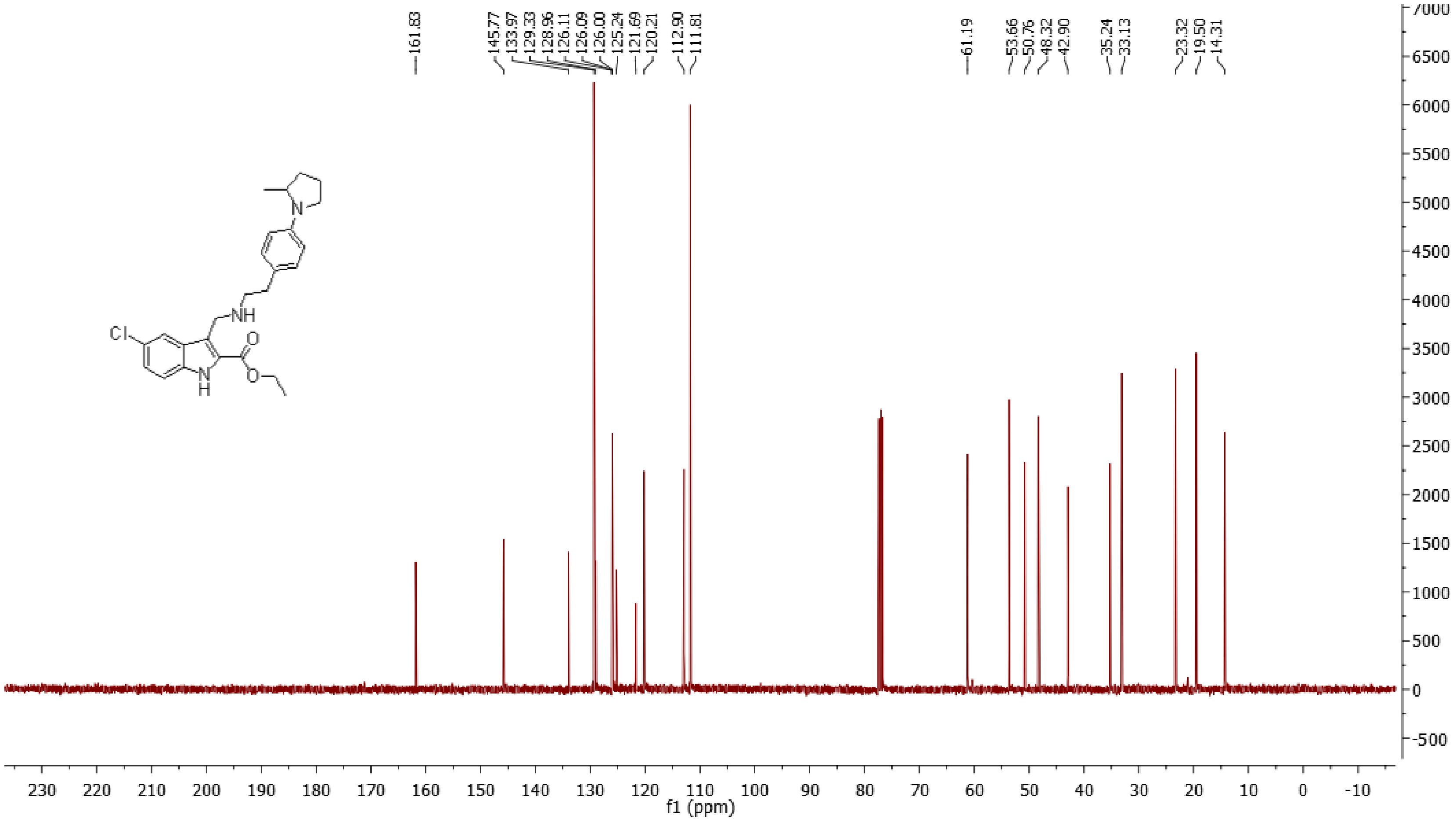

^{13}C NMR (101 MHz, cdcl$_3$) δ 161.83, 145.77, 133.97, 129.33, 128.96, 126.11, 126.09, 126.00, 125.24, 121.69, 120.21, 112.90, 111.81, 61.19, 53.66, 50.76, 48.32, 42.90, 35.24, 33.13, 23.32, 19.50, 14.31.

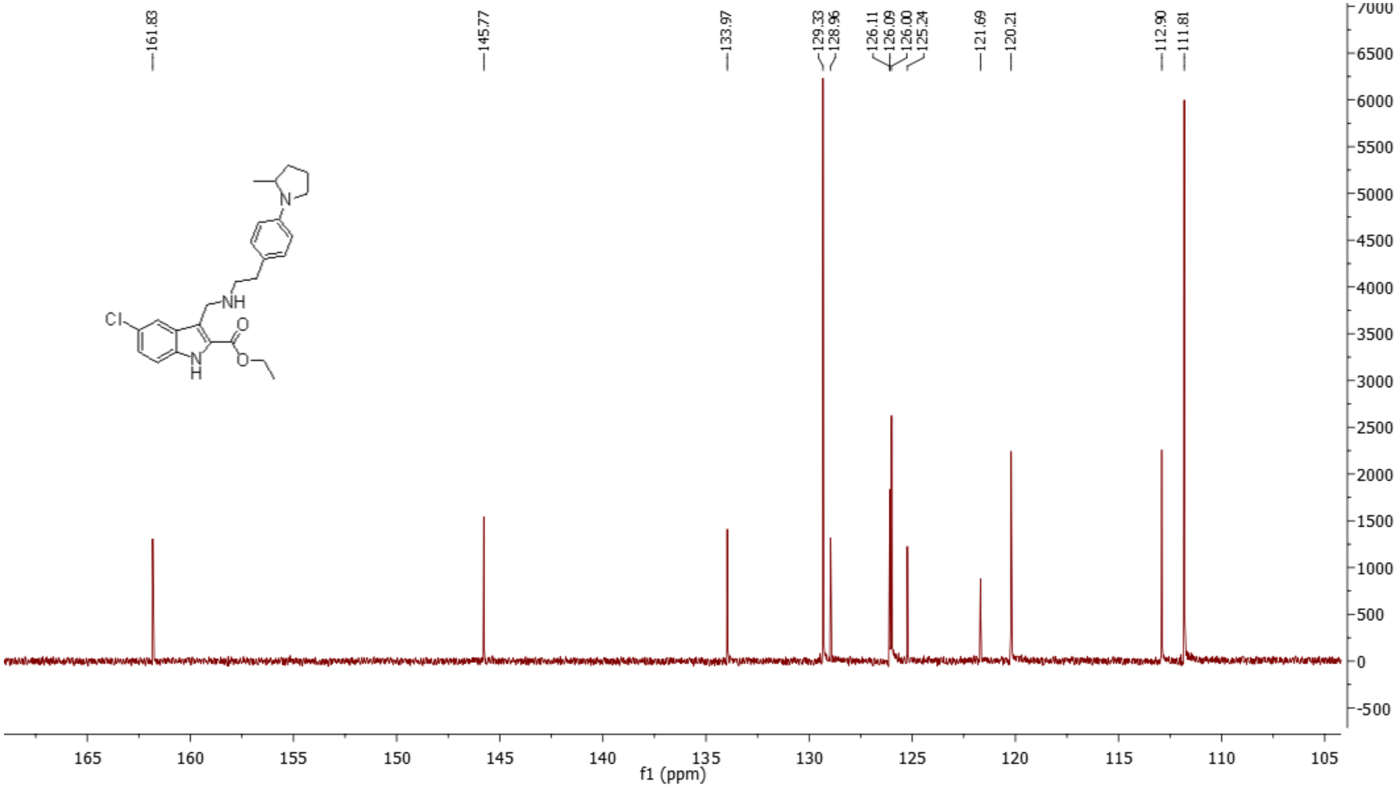

^{13}C NMR (101 MHz, cdcl$_3$) δ 161.83, 145.77, 133.97, 129.33, 128.96, 126.11, 126.09, 126.00, 125.24, 121.69, 120.21, 112.90, 111.81, 61.19, 53.66, 50.76, 48.32, 42.90, 35.24, 33.13, 23.32, 19.50, 14.31.

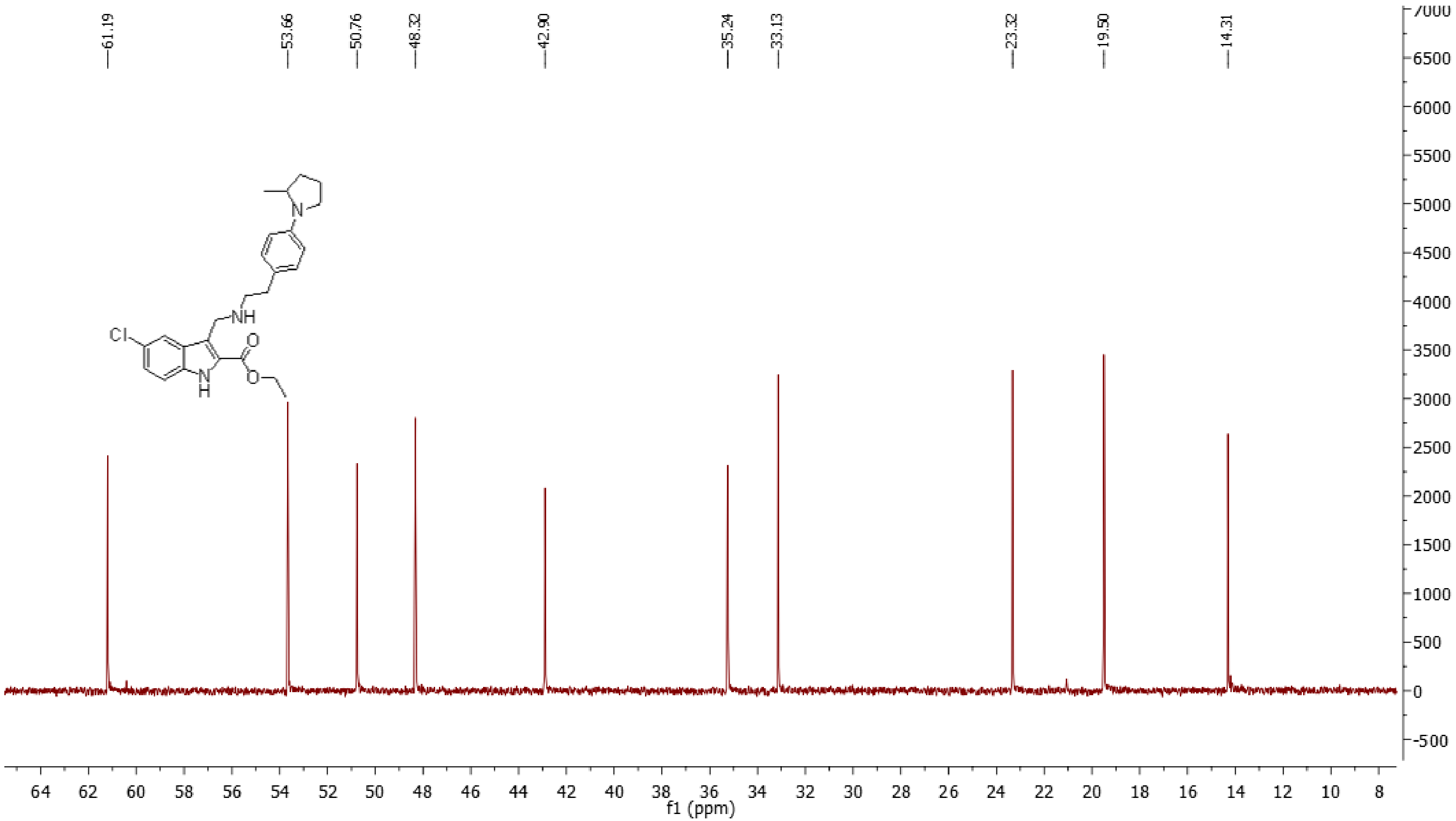

^{13}C NMR (101 MHz, cdcl$_3$) δ 161.83, 145.77, 133.97, 129.33, 128.96, 126.11, 126.09, 126.00, 125.24, 121.69, 120.21, 112.90, 111.81, 61.19, 53.66, 50.76, 48.32, 42.90, 35.24, 33.13, 23.32, 19.50, 14.31.

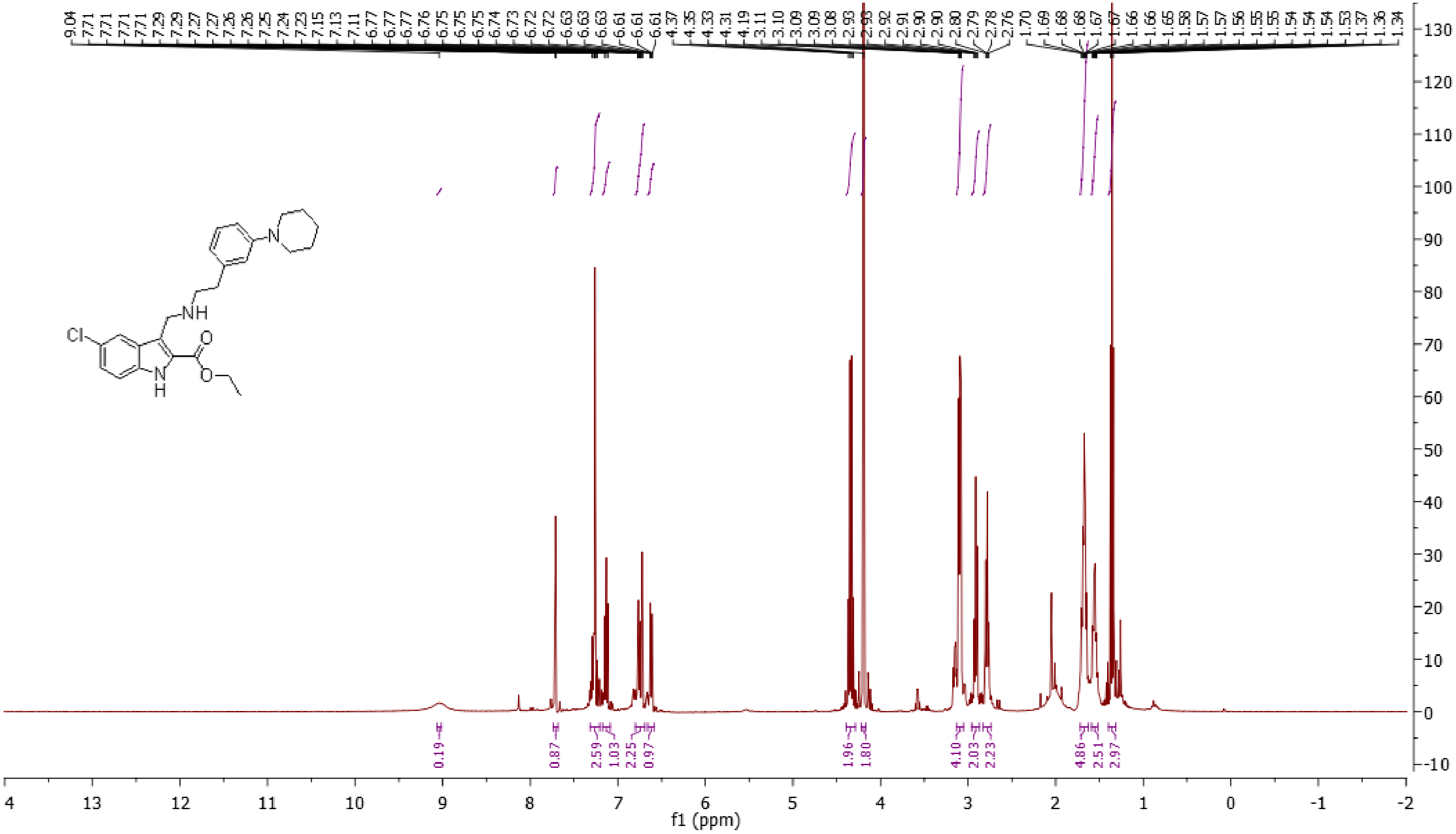

^{1}H NMR (400 MHz, Chloroform-*d*) δ 9.04 (s, 1H), 7.71 (d, *J* = 1.8 Hz, 1H), 7.32 – 7.21 (m, 2H), 7.13 (t, *J* = 7.8 Hz, 1H), 6.80 – 6.69 (m, 2H), 6.62 (d, *J* = 7.5 Hz, 1H), 4.34 (q, *J* = 7.1 Hz, 2H), 4.19 (s, 2H), 3.13 – 3.05 (m, 4H), 2.91 (t, *J* = 7.1 Hz, 2H), 2.78 (t, *J* = 7.2 Hz, 2H), 1.72 – 1.63 (m, 4H), 1.59 – 1.52 (m, 2H), 1.36 (t, *J* = 7.1 Hz, 3H).

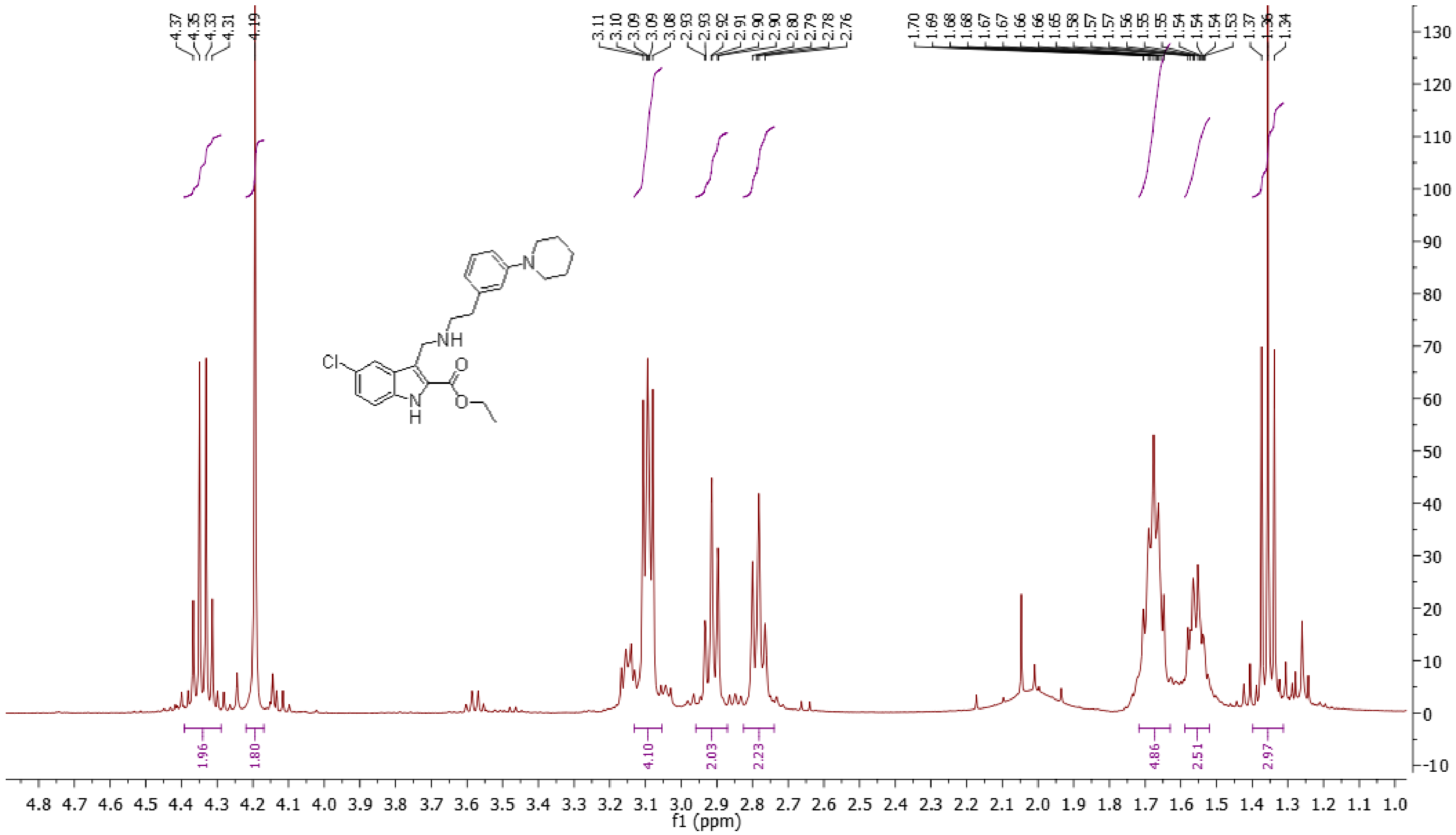

^{1}H NMR (400 MHz, Chloroform-*d*) δ 9.04 (s, 1H), 7.71 (d, *J* = 1.8 Hz, 1H), 7.32 – 7.21 (m, 2H), 7.13 (t, *J* = 7.8 Hz, 1H), 6.80 – 6.69 (m, 2H), 6.62 (d, *J* = 7.5 Hz, 1H), 4.34 (q, *J* = 7.1 Hz, 2H), 4.19 (s, 2H), 3.13 – 3.05 (m, 4H), 2.91 (t, *J* = 7.1 Hz, 2H), 2.78 (t, *J* = 7.2 Hz, 2H), 1.72 – 1.63 (m, 4H), 1.59 – 1.52 (m, 2H), 1.36 (t, *J* = 7.1 Hz, 3H).

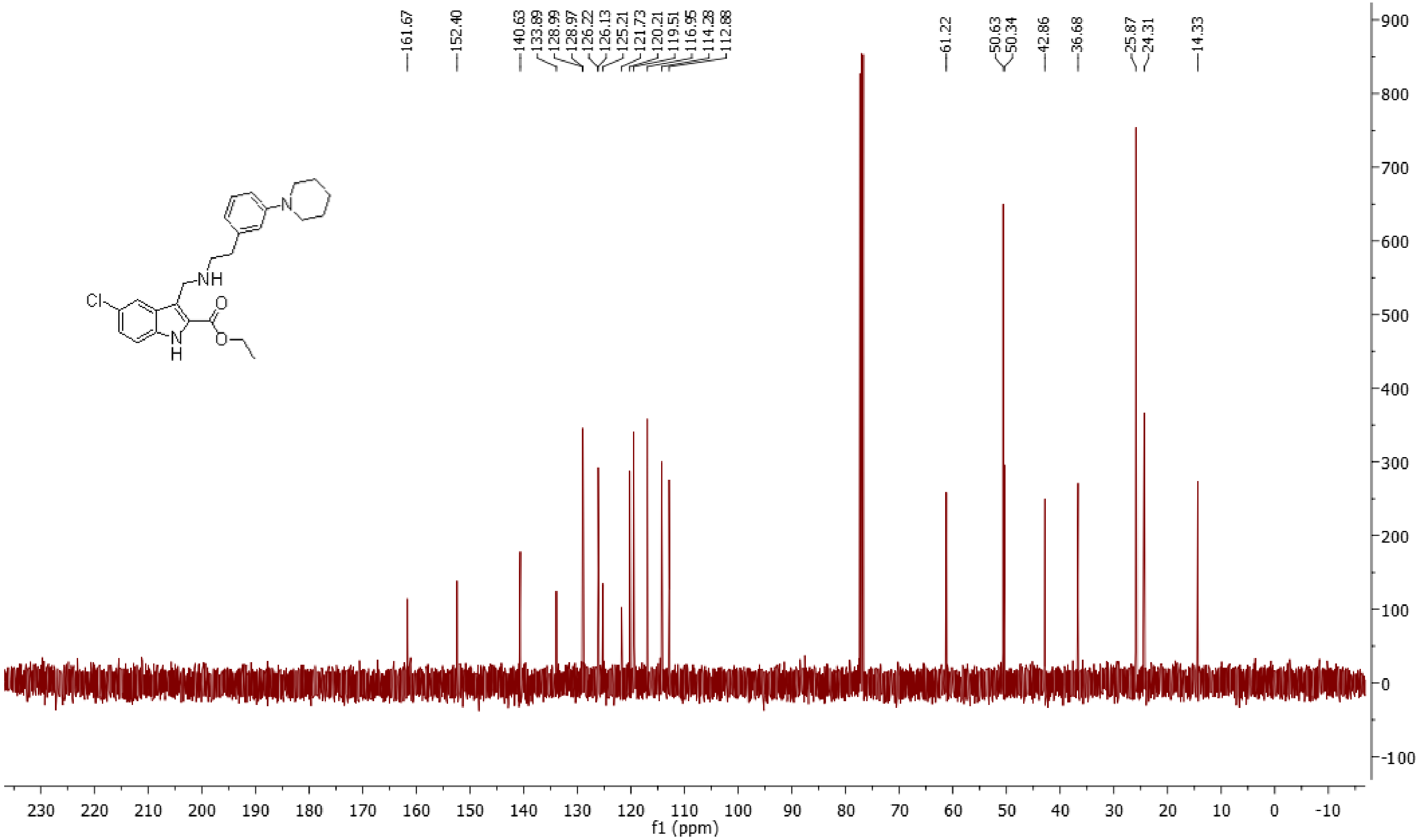

^{13}C NMR (101 MHz, cdcl$_3$) δ 161.67, 152.40, 140.63, 133.89, 128.99, 128.97, 126.22, 126.13, 125.21, 121.73, 120.21, 119.51, 116.95, 114.28, 112.88, 61.22, 50.63, 50.34, 42.86, 36.68, 25.87, 24.31, 14.33.

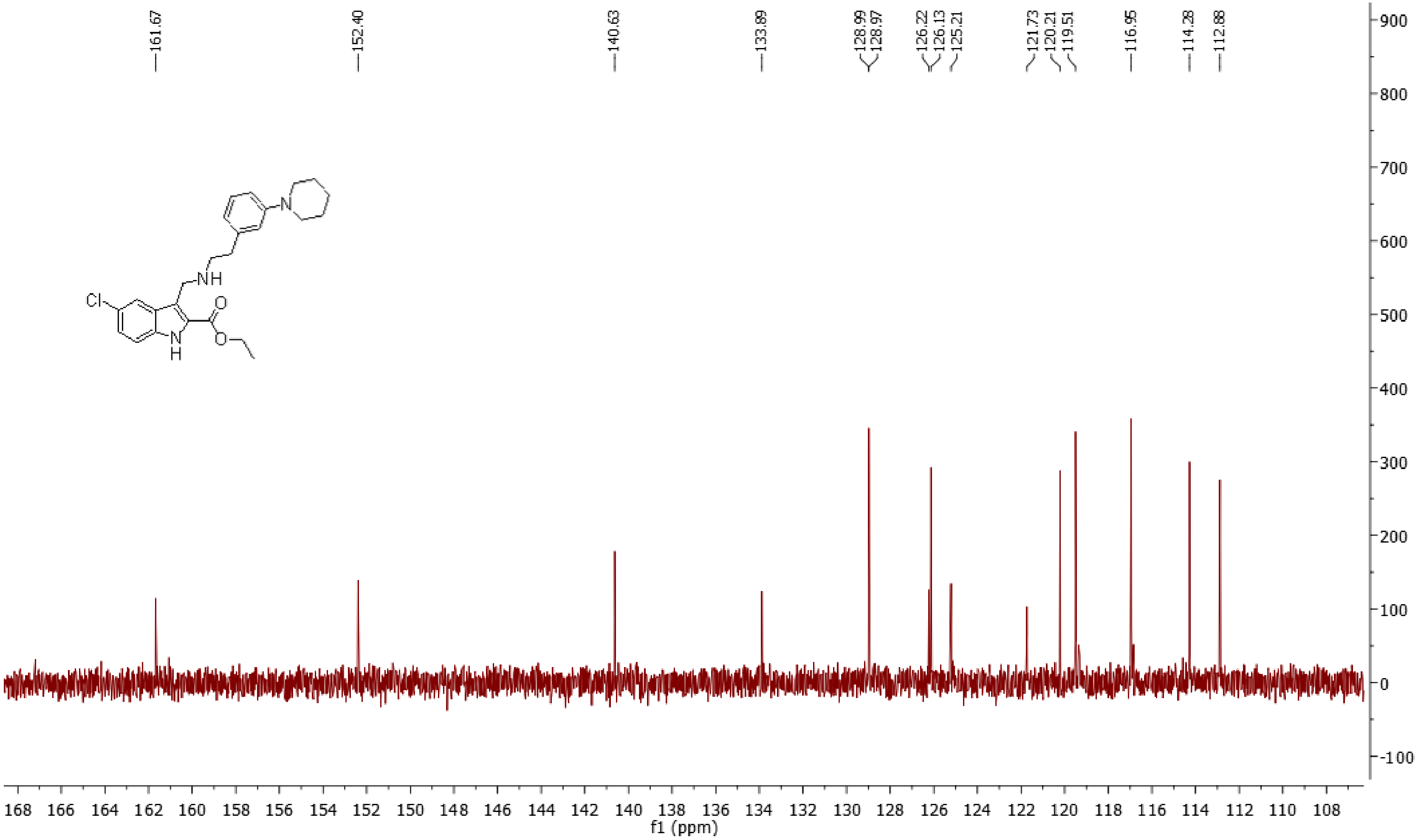

^{13}C NMR (101 MHz, cdcl$_3$) δ 161.67, 152.40, 140.63, 133.89, 128.99, 128.97, 126.22, 126.13, 125.21, 121.73, 120.21, 119.51, 116.95, 114.28, 112.88, 61.22, 50.63, 50.34, 42.86, 36.68, 25.87, 24.31, 14.33.

Compound 1 HRMS

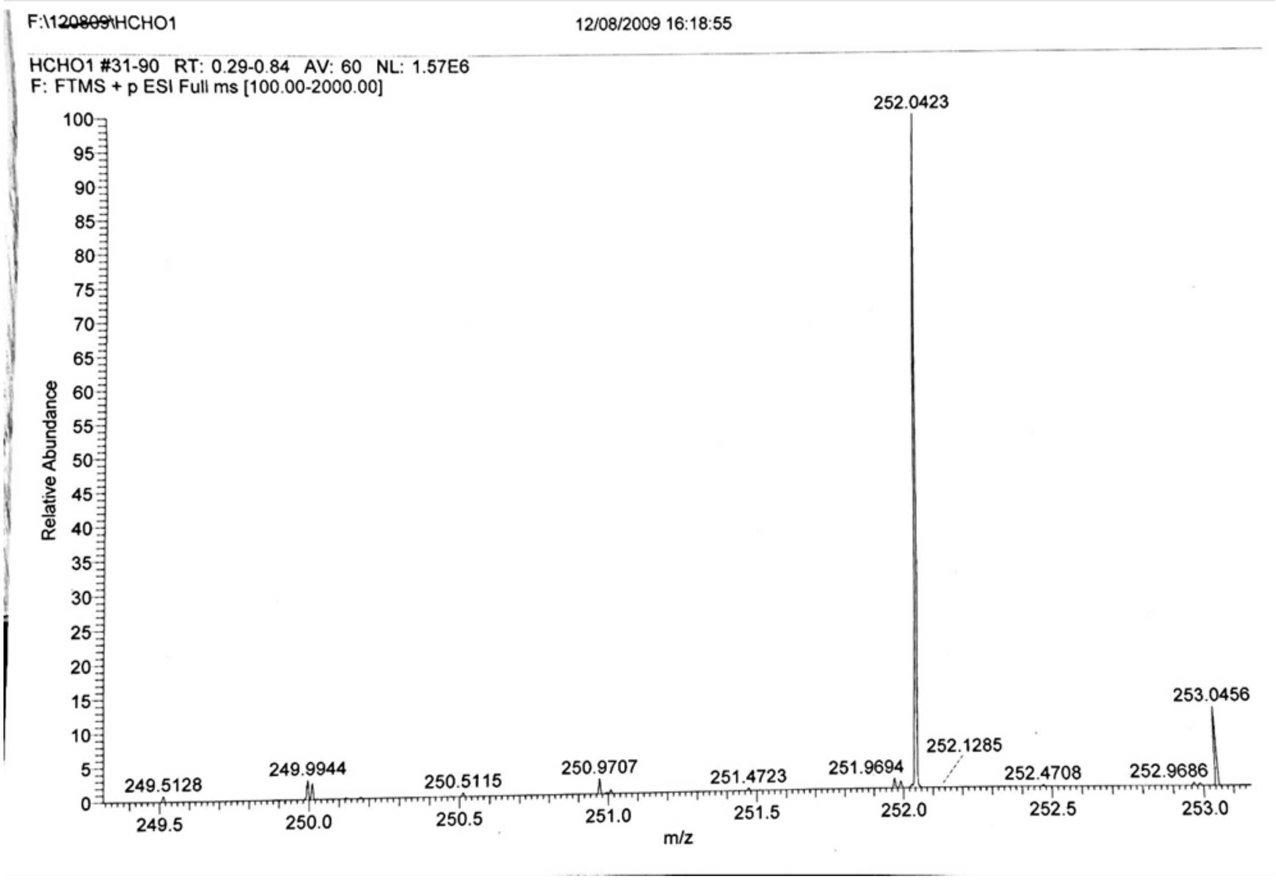

Compound 3a HRMS

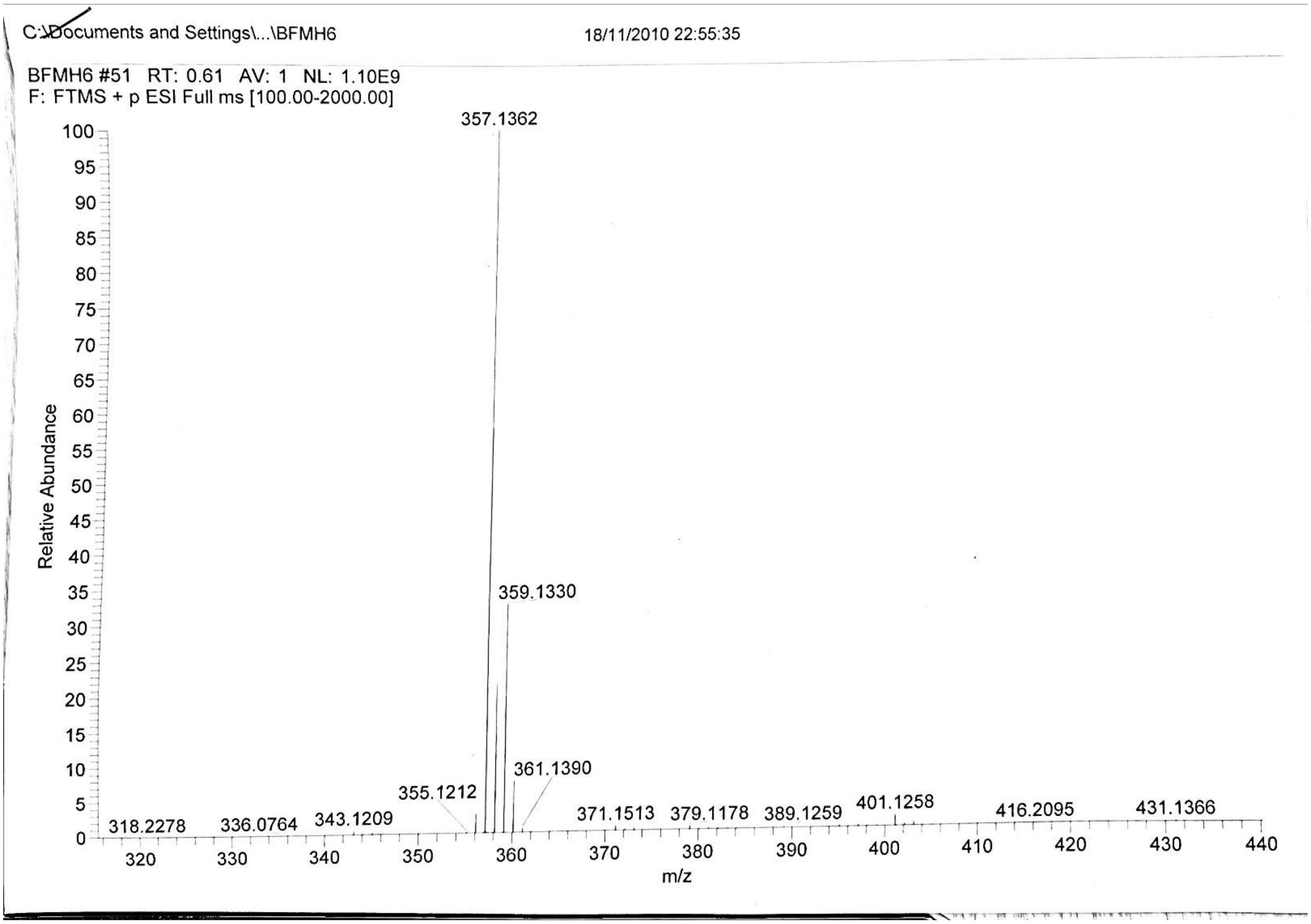

Compound 3b HRMS

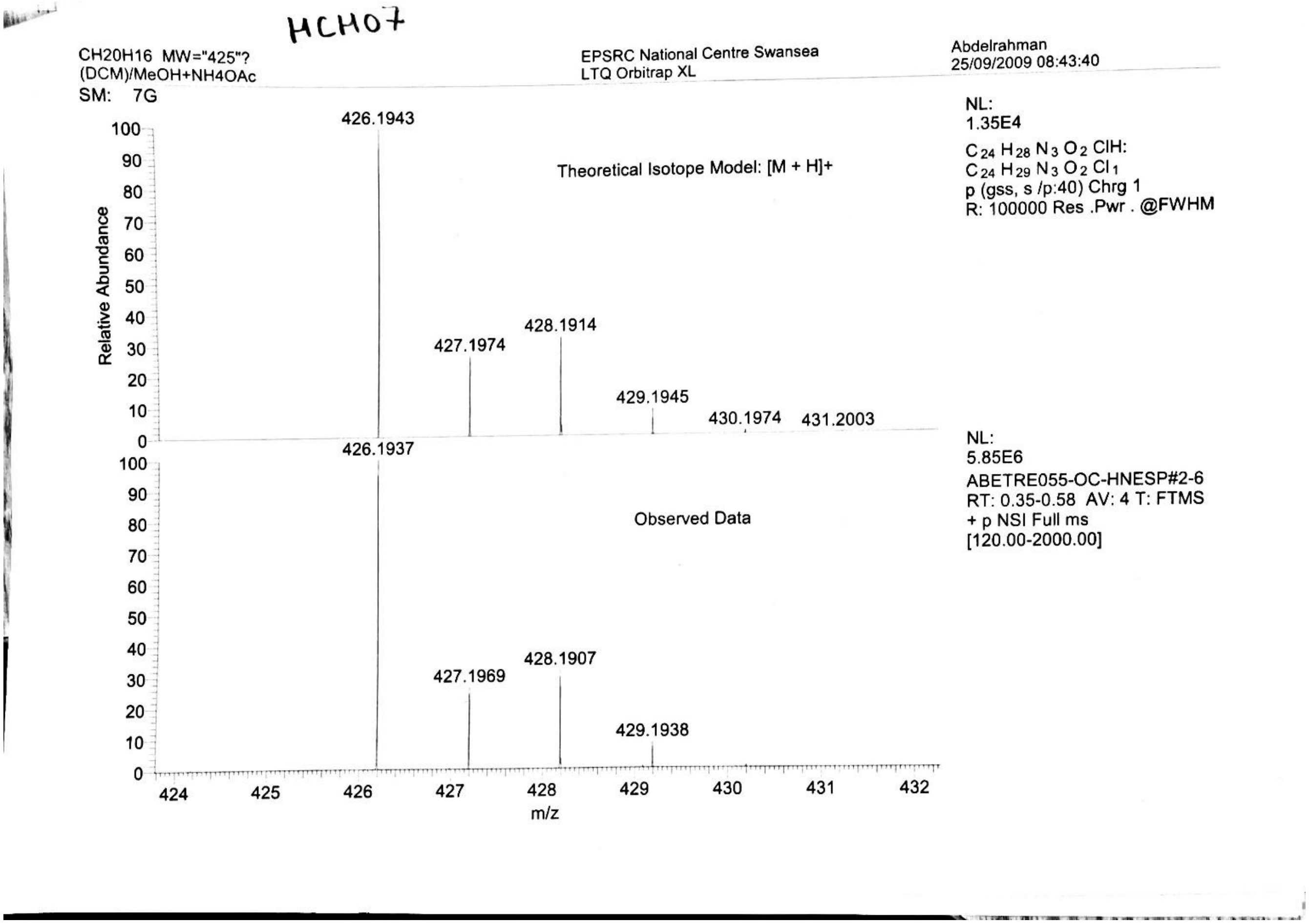

Compound 3c HRMS

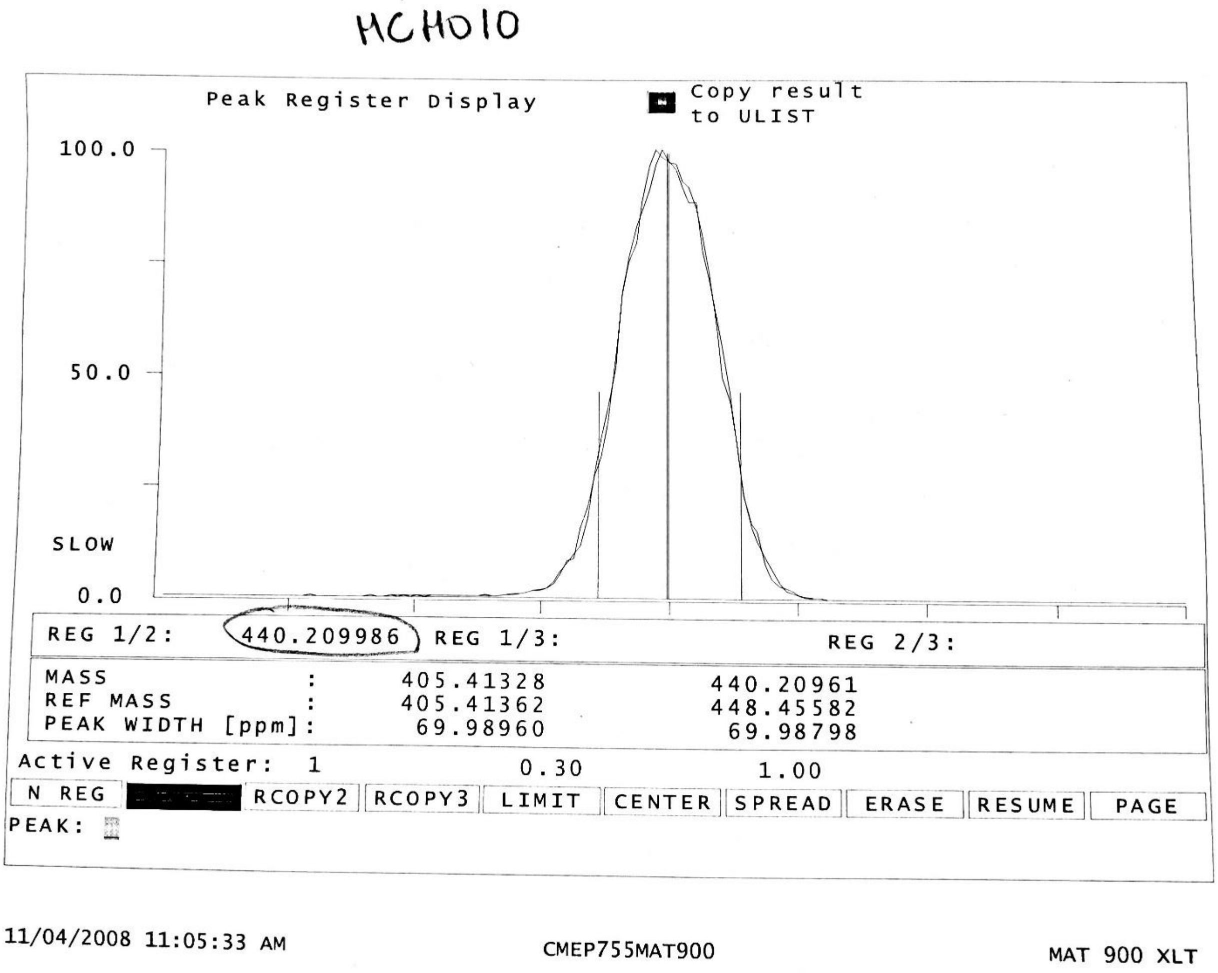

Compound 3d HRMS

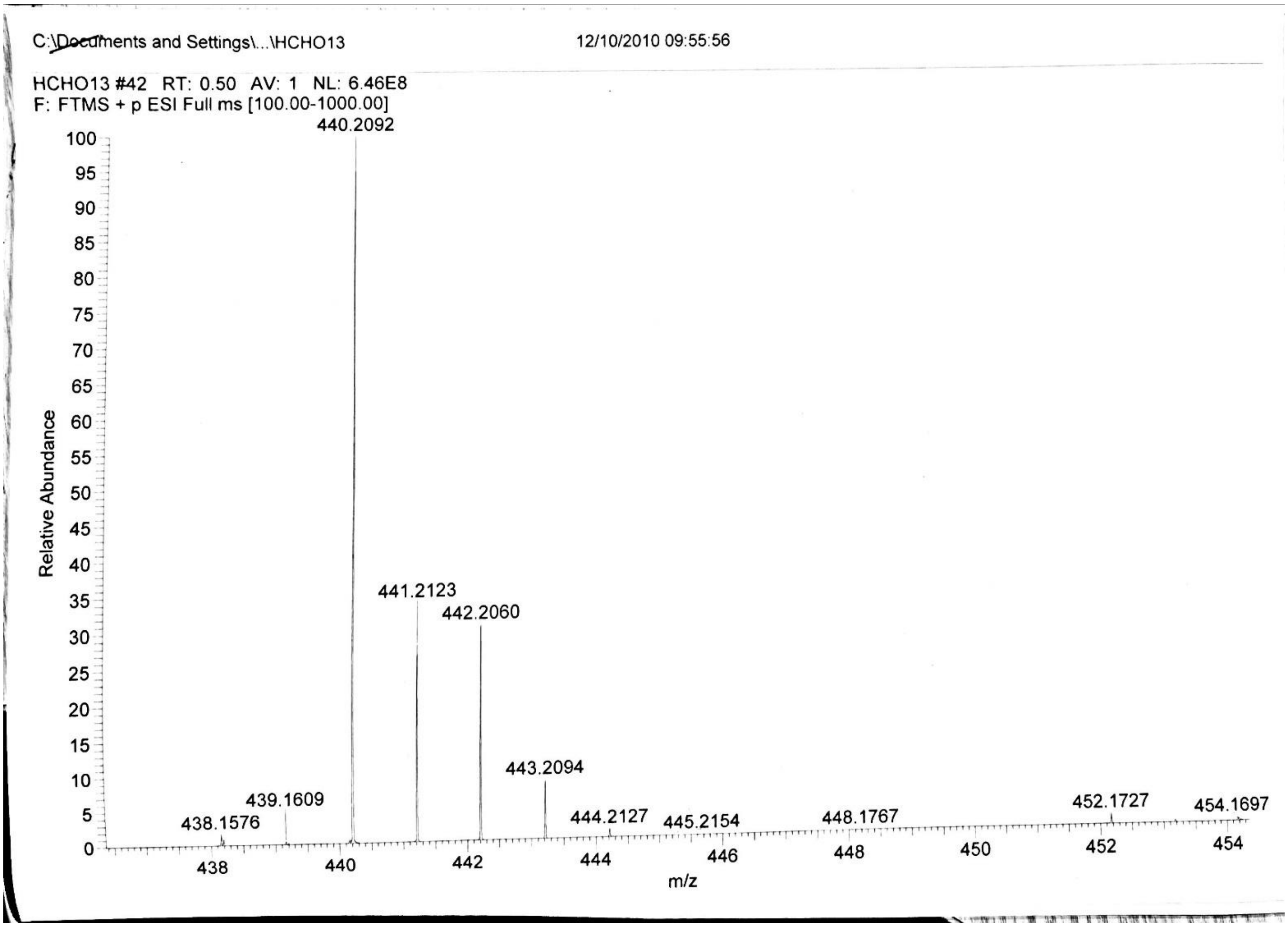

Compound 3e HRMS

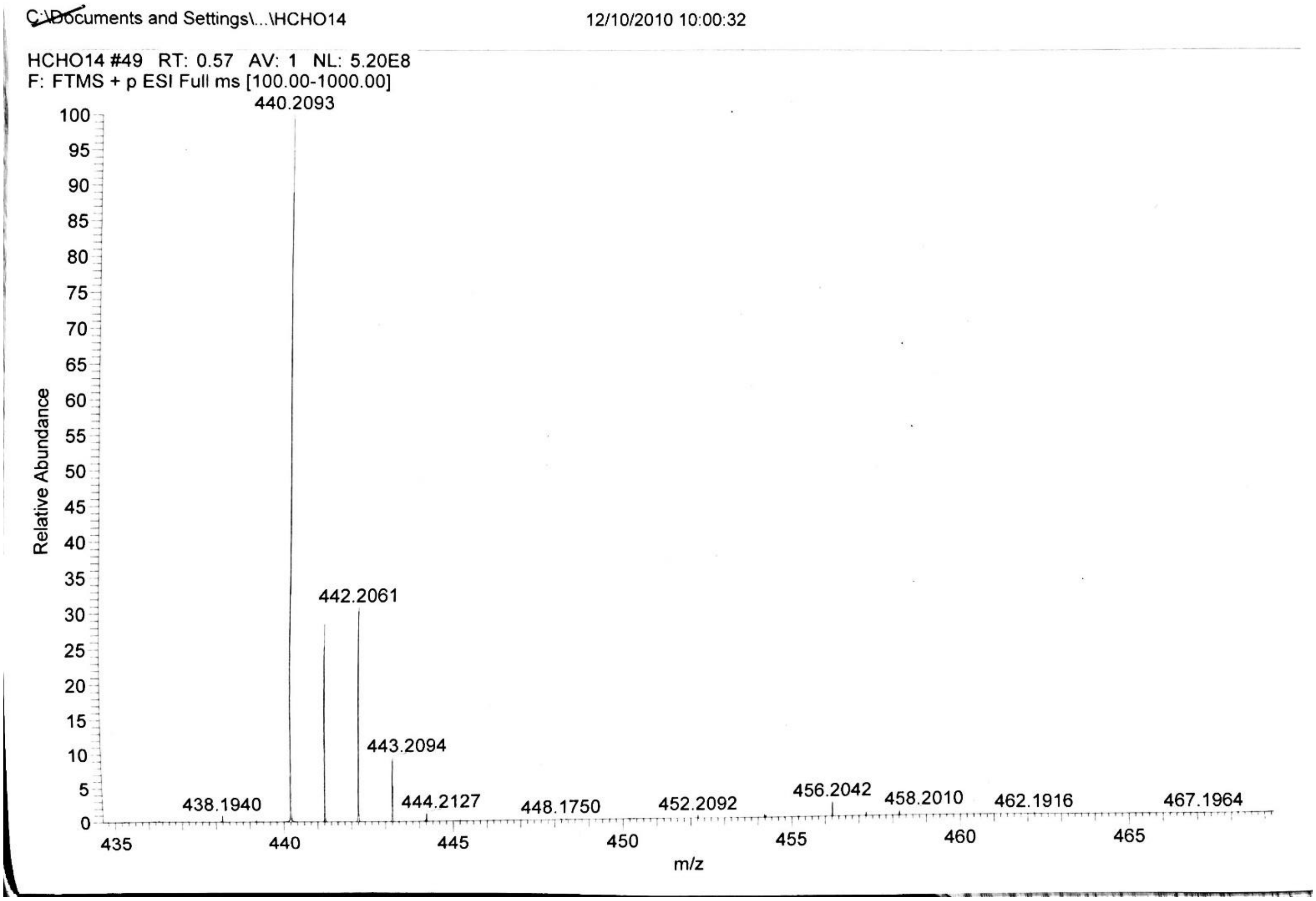

Compound 4a HRMS

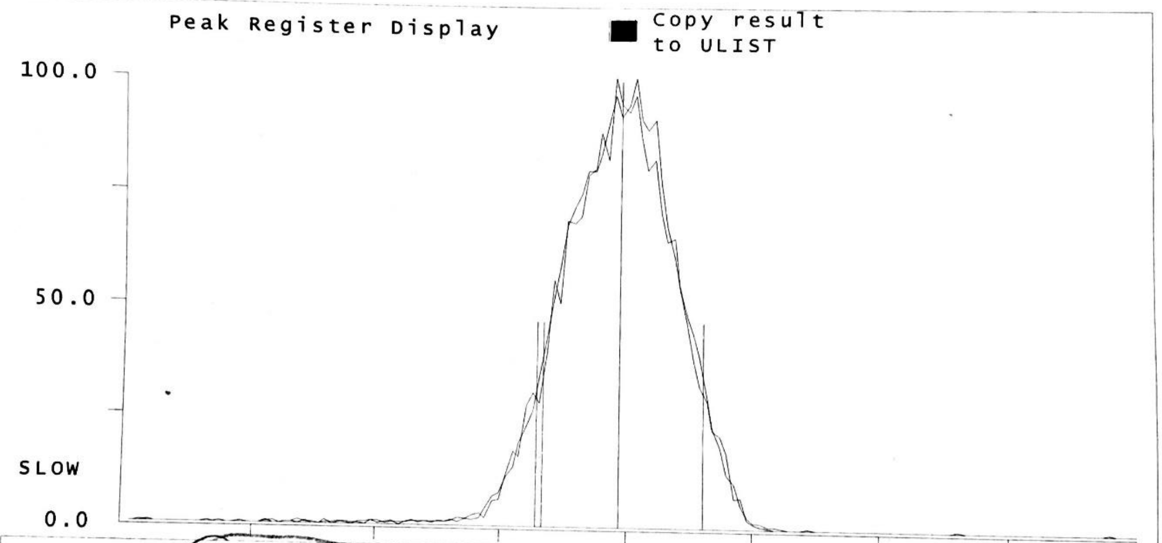

REG 1/2: 329.105247 REG 1/3: REG 2/3:

		REG 1/3	REG 2/3
MASS	:	319.32799	329.10398
REF MASS	:	319.32922	319.32922
PEAK WIDTH [ppm]	:	83.32070	79.99500

Active Register: 1 0.30 1.00

N REG | RCOPY2 | RCOPY3 | LIMIT | CENTER | SPREAD | ERASE | RESUME | PAGE

PEAK:

06/03/2008 9:14:33 PM CMEP755MAT900 MAT 900 XLT

Compound 4c HRMS

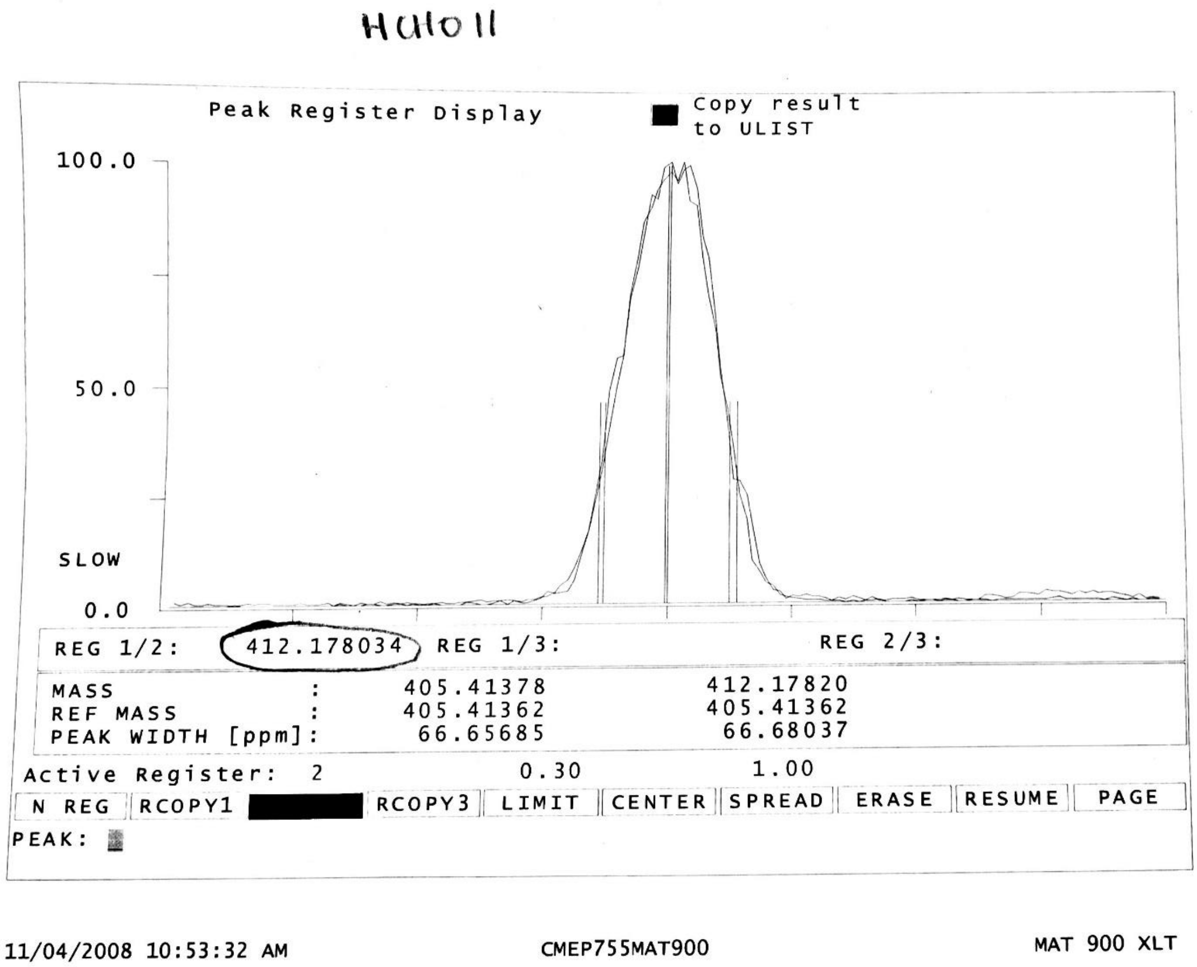

Compound 5b HRMS

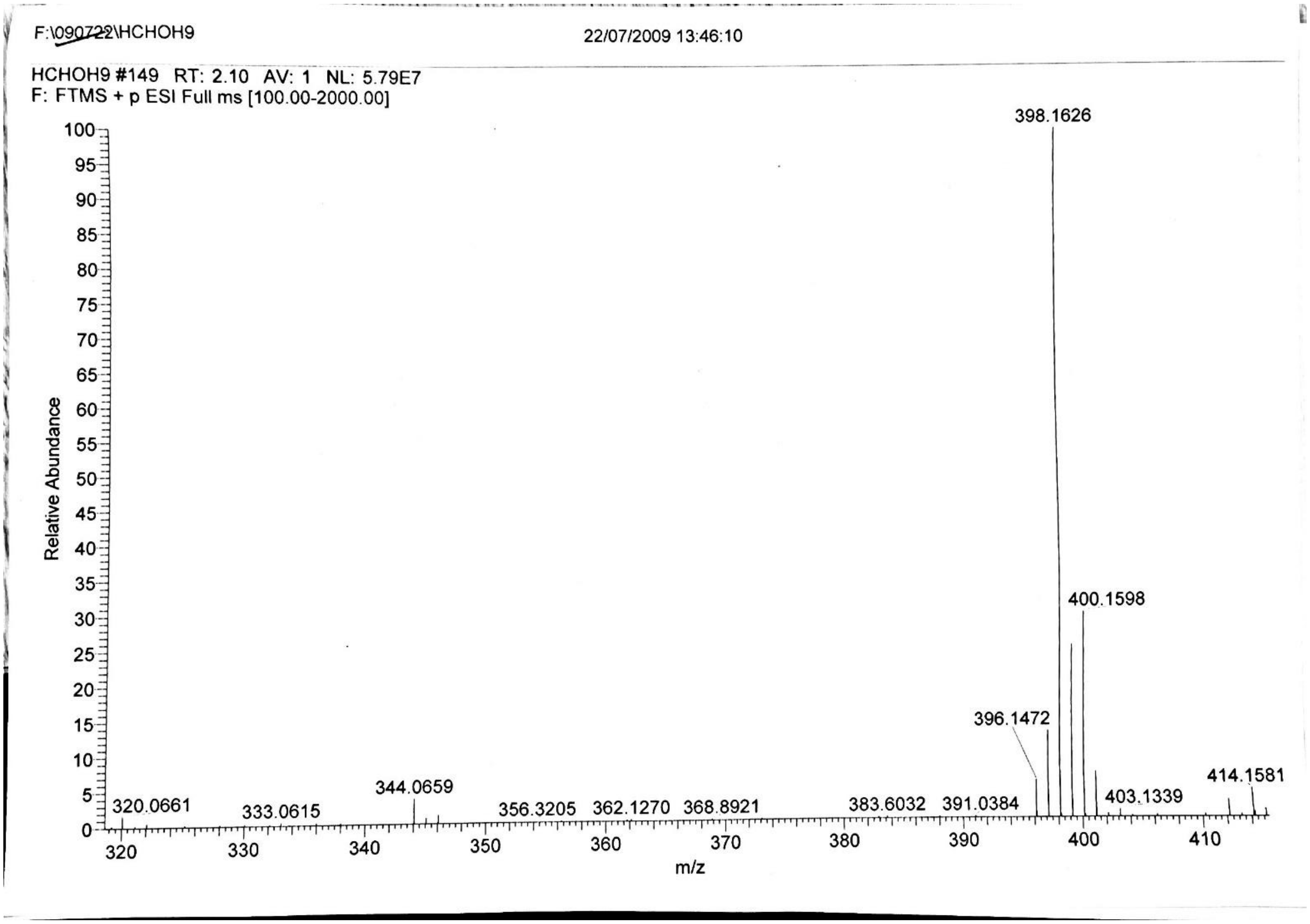

Compound 5c HRMS

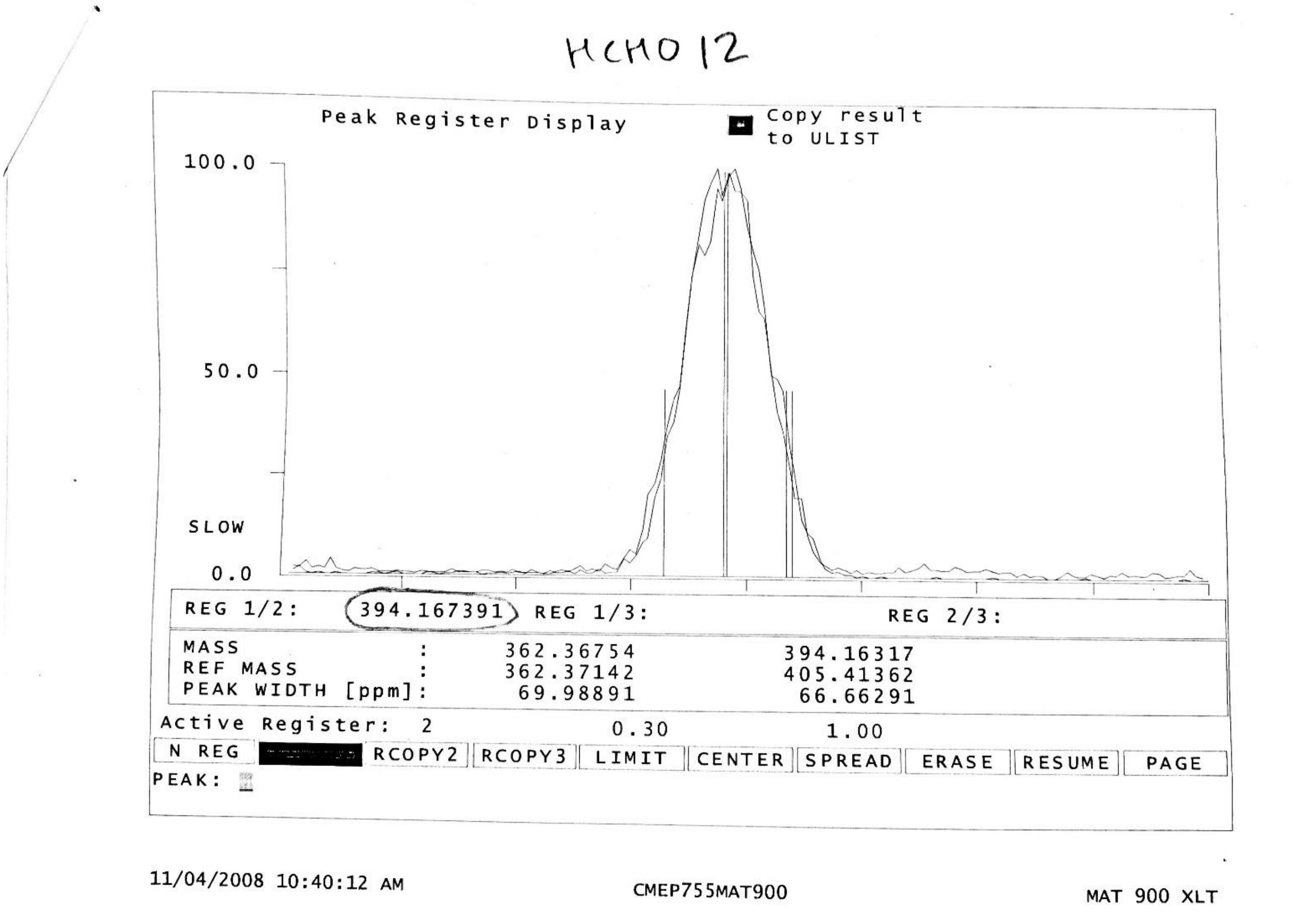

Appendix A

Experimental procedures

General Details

All the chemicals used were of analytical grade and purified by standard methods prior to use. Silica gel column chromatography was carried out using kieselgel 60 (Merck). TLC analysis was performed on aluminium-backed plates coated with silica gel 60 F_{254} (Merck). Melting points were determined using a Gallen Kamp melting point apparatus and are uncorrected. Components were visualized using potassium permanganate solution and UV light. NMR Spectra were taken using a Varian Unity INOVA 400 MHz and Bruker AC250 MHz spectrometers for proton and carbon at university of Aberdeen. All numbers referring to NMR data obtained are in parts per million (ppm). High resolution mass spectrometric data were obtained using Thermo Instruments MS system (LTQ XL/LTQ Orbitrap Discovery) coupled to a Thermo Instruments HPLC system (Accela PDA detector, Accela PDA autosampler and Pump) at university of Aberdeen

4.2. Biological evaluation

4.2.1. Cytotoxic activity using MTT Assay and evaluation of IC_{50}

4.2.1.1. MTT assay

MTT assay was performed to investigate the effect of the synthesized compounds on mammary epithelial cells (MCF-10A). The cells were propagated in medium consisting of Ham's F-12 medium/ Dulbecco's modified Eagle's medium (DMEM) (1:1) supplemented with 10% foetal calf serum, 2 mM glutamine, insulin (10 µg/mL), hydrocortisone (500 ng/mL) and epidermal growth factor (20 ng/mL). Trypsin

ethylenediamine tetra acetic acid (EDTA) was used to passage the cells after every 2-3 days. 96-well flat-bottomed cell culture plates were used to seed the cells at a density of 104 cells mL^{-1}. The medium was aspirated from all the wells of culture plates after 24 h followed by the addition of synthesized compounds (in 200 μL medium to yield a final concentration of 0.1% (v/v) dimethylsulfoxide) into individual wells of the plates. Four wells were designated to a single compound. The plates were allowed to incubate at 37°C for 96 h. Afterwards, the medium was aspirated and 3-[4,5-dimethylthiazol-2-yl]-2,5-diphenyltetrazolium bromide (MTT) (0.4 mg/mL) in medium was added to each well and subsequently incubated for 3 h. The medium was aspirated and 150 μL dimethyl sulfoxide (DMSO) was added to each well. The plates were vortexed followed by the measurement of absorbance at 540 nm on a microplate reader. The results were presented as inhibition (%) of proliferation in contrast to controls comprising 0.1% DMSO.

4.2.1.2. Assay for antiproliferative effect

To explore the antiproliferative potential of compounds propidium iodide fluorescence assay was performed using different cell lines such as Panc-1 (pancreas cancer cell line), MCF-7 (breast cancer cell line), HT-29 (colon cancer cell line) and A-549 (epithelial cancer cell line), respectively. To calculate the total nuclear DNA, a fluorescent dye (propidium iodide, PI) is used which can attach to the DNA, thus offering a quick and precise technique. PI cannot pass through the cell membrane and its signal intensity can be considered as directly proportional to quantity of cellular DNA. Cells whose cell membranes are damaged or have changed permeability are counted as dead ones. The assay was performed by seeding the cells of different cell lines at a density of 3000-7500 cells/well (in200μl medium) in culture plates followed by incubation for 24h at 37 °C in humidified 5%CO2/95% air atmospheric conditions.

The medium was removed; the compounds were added to the plates at 10 µM concentrations (in 0.1% DMSO) in triplicates, followed by incubation for 48h. DMSO (0.1%) was used as control. After incubation, medium was removed followed by the addition of PI (25 µl, 50µg/mL in water/medium) to each well of the plates. At -80 °C, the plates were allowed to freeze for 24 h, followed by thawing at 25° C. A fluorometer (Polar-Star BMG Tech) was used to record the readings at excitation and emission wavelengths of 530 and 620 nm for each well. The percentage cytotoxicity of compounds was calculated using the following formula:

$$\% \text{ Cytotoxicity} = \frac{A_C - A_{TC}}{A_C} \times 100$$

Where A*TC*= Absorbance of treated cells and AC= Absorbance of control. Erlotinib was used as positive control in the assay.

4.2.2. EGFR inhibitory assay

Baculoviral expression vectors including pBlueBacHis2B and pFASTBacHTc were used separately to clone 1.6 kb cDNA coding for EGFR cytoplasmic domain (EGFR-CD, amino acids 645–1186). 5′ upstream to the EGFR sequence comprised a sequence that encoded (His)6. Sf-9 cells were infected for 72h for protein expression. The pellets of Sf-9 cells were solubilized in a buffer containing sodium vanadate (100 µM), aprotinin (10 µg/mL), triton (1%), HEPES buffer(50mM), ammonium molybdate (10 µM), benzamidine HCl (16 µg/mL), NaCl (10 mM),leupeptin (10 µg/mL) and pepstatin (10 µg/mL) at 0°C for 20 min at pH 7.4, followed by centrifugation for 20 min. To eliminate the nonspecifically bound material, a Ni-NTA super flow packed column was used to pass through and wash the crude extract supernatant first with 10mM and then with 100 mM imidazole. Histidine-linked proteins were first eluted with 250 and then

with 500 mM imidazole after dialysis against NaCl (50 mM), HEPES (20 mM), glycerol (10%) and 1 µg/mL each of aprotinin, leupeptin and pepstatin for 120 min. The purification was performed either at 4 °C or on ice. To record autophosphorylation level, EGFR kinase assay was carried out based on DELFIA/Time-Resolved Fluorometry. The compounds were first dissolved in DMSO absolute, after dilution to appropriate concentration using HEPES (25 mM) at pH 7.4. Each compound (10 µL) was incubated with recombinant enzyme (10 µL, 5 ng for EGFR, 1:80 dilution in 100 mM HEPES) for 10 min at 25°C, after the addition of 5X buffer (10 µL, containing 2 mM MnCl2, 100 µM Na3VO4, 20 mM HEPES and 1 mM DTT) and ATP-MgCl2 (20 µL, containing 0.1 mM ATP and 50 mM MgCl2) and incubation for 1h. The negative and positive controls were included in each plate by the incubation of enzyme either with or without ATP-MgCl2. The liquid was removed after incubation and the plates were washed thrice using wash buffer. Europium-tagged antiphosphotyrosine antibody (75 µL, 400 ng) was added to each well followed by incubation of 1h and then washing of the plates using buffer. The enhancement solution was added to each well and the signal was recorded at excitation and emission wavelengths of 340 at 615 nm. The autophosphorylation percentage inhibition by compounds was calculated using the following equation:

$$100\% - [(negative\ control)/(positive\ control) - (negative\ control)]$$

Using the curves of percentage inhibition of eight concentrations of each compound, IC50 was calculated. Majority of signals detected by antiphosphotyrosine antibody were from EGFR because the enzyme preparation contained low impurities.

$EGFR^{T790M}$ inhibitory assay

The EGFR protein (EGFRT790M) was purchased from Carna Bioscience. The HTRF KinEASE-TK kit (Cat#62TK0PEB) was from Cisbio. All the other chemicals were from Sigma. The EGFR kinase assays were performed in 384-well plate (Corning 3676, low volume, black, NBS), using Cisbio HTRF KinEASE-TK kit. The assay buffer contained 50 mM HEPES (Ph 7.0), 0.02% NaN3, 0.01% BSA, 0.1 mM Orthovanadate, 10 mM MgCl2, 2.5 mM DTT and 6.25 nM SEB. EGFR kinases was first incubated with compounds for 2 h respectively, then ATP/Peptide substrate mixture was added to initiate the reaction. After 30 min reaction at room temperature, the detection reagents were added. The TR-FRET signal was measured on PerkinElmer Envision using excitation 320 nm and emission 615 nm/665 nm. The data was analyzed using Graph Pad Prism (Version 6.0) software after three parallel experiments.

BRAF kinase assay

V^{600E} mutant BRAF kinase assay was performed to investigate the activity of compounds **20-23**, **28-31** and **33** against BRAF. Mouse full-length GST-tagged $BRAF^{V600E}$ (7.5 ng, Invitrogen, PV3849) was pre-incubated with drug (1 µL) and assay dilution buffer (4 µL) for 60 min at 25°C. In assay dilution buffer, a solution (5 µL) containing $MgCl_2$ (30 mM), ATP (200 µM), recombinant human full length (200 ng) and *N*-terminal His-tagged MEK1 (Invitrogen) was added to start the assay, after incubation for 25 min at 25°C. The assay was stopped using 5X protein denaturing buffer (LDS) solution (5 µL). To further denature the protein, heat (70° C) was applied for 5 min. 4-12% precast NuPage gel plates (Invitrogen) were used to carry out electrophoresis (at 200 V). 10 µL of each reaction was loaded into the precast plates and electrophoresis was allowed to proceed. After completion of electrophoresis, the front part of the precast gel plate (holding hot ATP) was cut and afterwards cast-off.

The dried gel was developed using a phosphor screen. A reaction without active enzyme was used as negative control while that containing no inhibitor served as positive control. To study the effect of compounds on cell-based pERK1/2 activity in cancer cells, commercially available ELISA kits (Invitrogen) were used according to manufacturer's instructions.

Statistical analysis

Computerized Prism 5 program was used to statistically analyzed data using one-way ANOVA test followed by Tukey's as post ANOVA for multiple comparison at $P \leq .05$. Data were presented as mean ± SEM.
